# The amino acid transporter SLC7A11 expression in breast cancer

**DOI:** 10.1080/15384047.2023.2291855

**Published:** 2023-12-10

**Authors:** Preyanka Nath, Lutfi H. Alfarsi, Rokaya El-Ansari, Brendah K. Masisi, Busra Erkan, Ali Fakroun, Ian O. Ellis, Emad A. Rakha, Andrew R. Green

**Affiliations:** a Nottingham Breast Cancer Research Centre, Academic Unit of Translational Medical Sciences, School of Medicine, University of Nottingham Biodiscovery Institute, Nottingham, UK; b Cellular Pathology, Nottingham University Hospitals NHS Trust, Nottingham City Hospital, Nottingham, UK

**Keywords:** Breast cancer, subtypes, SLC7A11 expression, amino acid transporters, prognostic factor, IHC, metabolism

## Abstract

Breast cancer (BC), characterized by its diverse molecular profiles and clinical outcomes, presents a significant challenge in the development of effective therapeutic strategies. Metabolic reprogramming, a defining characteristic of cancer, has emerged as a promising target for novel therapies. SLC7A11, an amino acid transporter that facilitates cysteine uptake in exchange for glutamate, plays a crucial role in sustaining the altered metabolism of cancer cells. This study delves into the comprehensive analysis of SLC7A11 at the genomic, transcriptomic, and protein levels in extensive BC datasets to elucidate its potential role in different BC subtypes. *SLC7A11* gene copy number and mRNA expression were evaluated using the Molecular Taxonomy of Breast Cancer International Consortium (METABRIC) cohort (*n* = 1,980) and Breast Cancer Gene Expression Miner (*n* = 4,712). SLC7A11 protein was assessed using immunohistochemistry in a large BC cohort (*n* = 1,981). Additionally, The Cancer Genome Atlas (TCGA) dataset was used to explore *SLC7A11* DNA methylation patterns using MethSurv (*n* = 782) and association of *SLC7A11* mRNA expression with immune infiltrates using TIMER (*n* = 1,100). High *SLC7A11* mRNA and SLC7A11 protein expression were significantly associated with high tumor grade (*p* ≤ .02), indicating a potential role in cancer progression. Interestingly, SLC7A11 copy number gain was observed in HER2+ tumors (*p* = .01), suggesting a subtype-specific association. In contrast, SLC7A11 mRNA expression was higher in the basal-like/triple-negative (TN; *p* < .001) and luminal B tumors (*p* = .02), highlighting its differential expression across BC subtypes. Notably, high SLC7A11 protein expression was predominantly observed in Estrogen Receptor (ER)-negative and Triple Negative (TN) BC, suggesting a role in these aggressive subtypes. Further analysis revealed that SLC7A11 was positively correlated with other amino acid transporters and enzymes associated with glutamine metabolism, implying a coordinated role in metabolic regulation. Additionally, *SLC7A11* gene expression was positively associated with neutrophil and macrophage infiltration, suggesting a potential link between SLC7A11 and tumor immunity. Our findings suggest that SLC7A11 plays a significant role in BC metabolism, demonstrating differential expression across subtypes and associations with poor patient outcomes. Further functional studies are warranted to elucidate the precise mechanisms by which SLC7A11 contributes to BC progression and to explore its potential as a therapeutic target.

## Introduction

Breast cancer (BC), the top malignancy in women,^1^ is a heterogeneous disease with various biological subtypes that differ in morphology, biological subtypes, response to therapy and clinical behavior.^2^ This variability further underscores the notion of varying cancer hallmarks and drivers within the different subtypes of breast cancer.

Metabolic reprogramming ensures tumors have a sufficient supply of nutrients to support their rapid growth and proliferation, playing a central role in energy production and biosynthesis.^3^ This altered metabolism likely contributes to tumor progression and therapeutic failure, which further reinforces the importance of novel therapeutic strategies targeting metabolic reprogramming. Glutamine metabolism is a particular focus of attention as cancer cells can be heavily dependent on this most abundant, non-essential amino acid for nutritional uptake fueling cancer cell unremitted growth.^4^ Glutamine metabolism and associated metabolic networks including expression of related amino acid transporters are essential for cancer cell survival.^5^ Rapidly proliferating cancer cells take up glutamine via these amino acid transporters and subsequently transform it into glutamate by glutaminase, a process called glutaminolysis.^6^ This intracellular glutamate is exchanged with extracellular cystine, the oxidized dimer form of cysteine, at a ratio of 1:1 through Solute Carrier Family 7 Member 11 (SLC7A11) which is later converted into cysteine. SLC7A11 is a sodium independent, chloride-dependent anionic L-cystine/L-glutamate antiporter on the cell surface.^7^ Intracellular cysteine is generally produced *de novo* or re-utilized through protein degradation.^8–10^ However, in oxidative stress, *de novo* biosynthesis or a catabolic supply of cysteine is not sufficient to meet the high requirement for antioxidant synthesis by cancer cells. Hence, most rely on amino acid transporters, such as SLC7A11, that can import extracellular cystine^8^ and thus metabolic reprogramming orchestrates the energy supply reflecting the altered metabolic needs of tumors.^11^

SLC7A11 mediated cystine uptake plays a positive but rate limiting role in glutathione synthesis, contributing to the pathogenesis of cancer where glutathione activates the antioxidant defense machinery that protects cancer cells from apoptosis, oxidative stress and ferroptosis.^7,9,12,13^ SLC7A11 is expressed at a low level in normal cells but remains a major transporter for cancer cells that are largely reliant on extracellular cystine for survival,^10^ including liver, glioma, and lung cancer directing disease survival.^8,12,14–23^ The opposing expression levels between normal and cancer cell suggest the SLC7A11 could act as a potential target for cancer treatment. In BC, SLC7A11 is suggested to play a crucial role in cancer stem cells impacting metastasis.^24^ In TNBC, the most aggressive biological subtype with poor survival, it also plays an important role in disease progression and proliferation.^25,26^

Given the diverse origins and divergent disease progression of breast cancer (BC) subtypes, these subtypes are likely to possess distinct genomic, transcriptomic, and proteomic alterations that influence clinical outcomes and treatment responses. Metabolic heterogeneity is also evident among BC biological subtypes, with differential expression of glutamine metabolism-related proteins, a phenomenon that may be driven by ER expression.^27–29^ Therefore, it is hypothesized that SLC7A11 expression also exhibits heterogeneity in BC, leading to varying outcomes across different subtypes. This prompted a comprehensive investigation to assess the impact of SLC7A11 in BC, evaluating its transcriptomic and proteomic expression in BC subtypes as a potential prognostic marker.

## Results

### SLC7A11 expression in breast cancer

High *SLC7A11* mRNA expression (log2 intensity > 5.5) was observed in 239/1,980 (12%) of the cases. Most tumors showed normal *SLCA11* gene copy number (CN) with only 23/1,980 (1.2%) cases showing *SLC7A11* CN gain whereas 66/1,980 (3.3%) cases had gene CN loss. No significant association was seen between *SLC7A11* gene CN variation (CNV) and mRNA expression (*p* = .180, data not shown).

**Figure 1.**
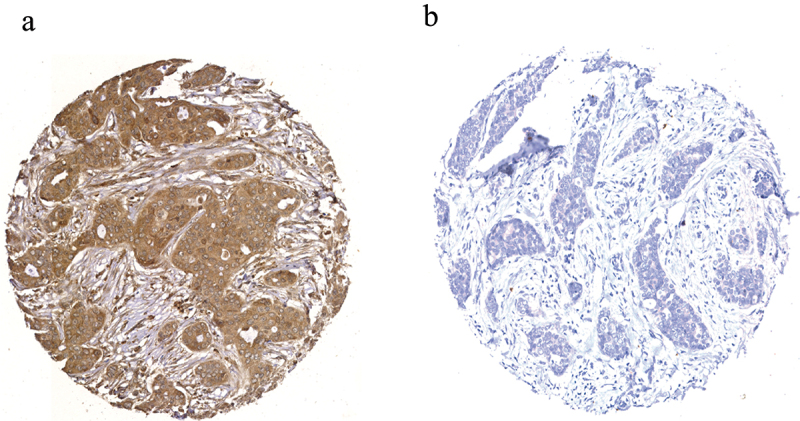
SLC7A11 protein expression in representative invasive breast cancer tissue microarray cores determined using immunohistochemistry showing a) high, and b) negative expression. Magnification x20.

Staining of SLC7A11 in full-face BC tissue sections demonstrated a homogenous pattern of immunoreactivity. SLC7A11 protein expression was mainly observed in the cytoplasm of the invasive breast tumor cells, with intensity levels varying from absent to high (Figure 1). The distribution of SLC7A11 expression was unimodal and left-skewed and X-tile software was used to identify the optimal cutoff point in predicting breast cancer specific survival (BCSS). High SLC7A11 protein (>100 H-score) was observed in 784/1,981 (40%) of cases.

### SLC7A11 *DNA methylation in breast cancer*

Several CpG islands showed high *SLC17A11* DNA methylation within the genomic body of the gene including cg21877274 and cg24869834 (Supplementary Figure S2). The first exon in the 5’ untranslated region showed little methylation.

### Association of SLC7A11 expression with clinicopathological characteristics

**Figure 2.**
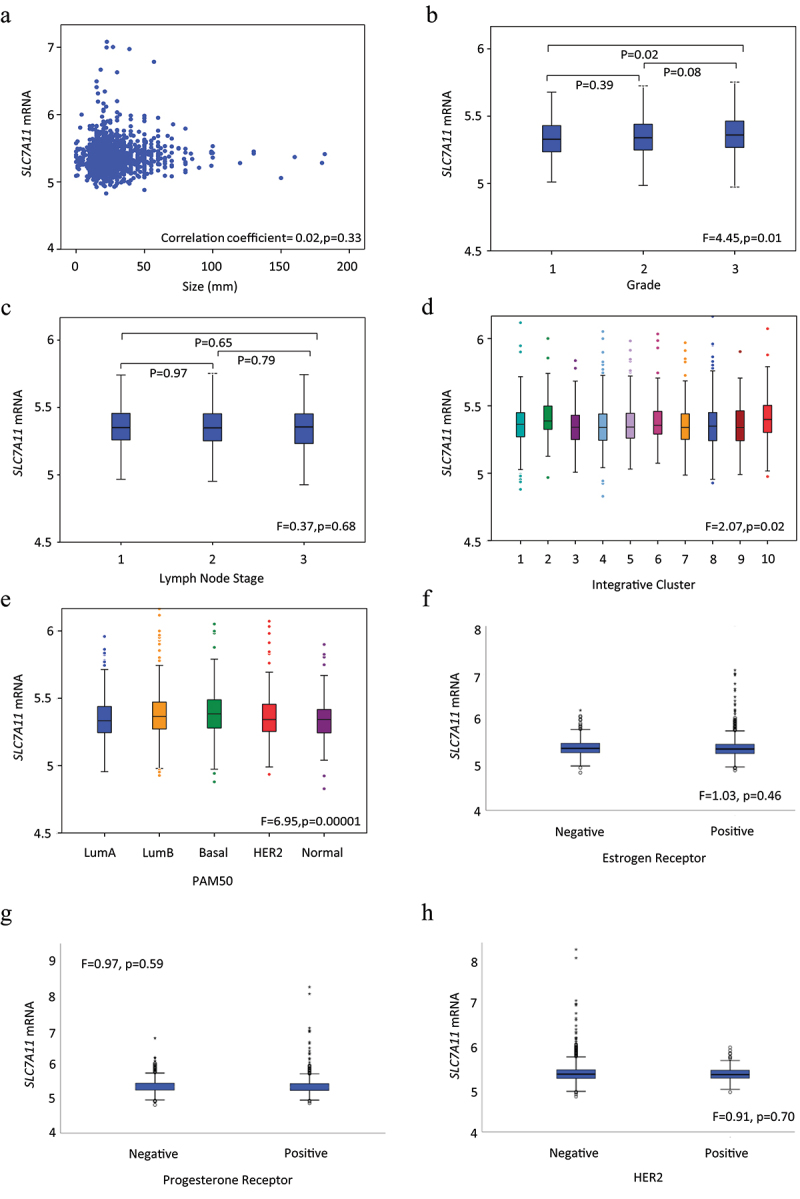
*SLC7A11* mRNA expression and its association with clinicopathological parameters and molecular subtypes in the METABRIC cohort: a) *SLC7A11* and tumor size, b) *SLC7A11* and tumor grade, c) *SLC7A11* and lymph node stage, d) *SLC7A11* and METABRIC Integrative clusters, e) *SLC7A11* and PAM50 subtypes, f) *SLC7A11* and ER, g) *SLC7A11* and PR, h) *SLC7A11* and HER2.

Within the METABRIC cohort, *SLC7A11* CN gain was significantly associated with invasive breast carcinoma of no special type (NST) (*p* = .0002; Table 1). High *SLC7A11* mRNA expression was associated with high tumor grade (*p* = .01, Figure 2b) but not with tumor size or nodal stage (Figure 2a,c). Within the Breast Cancer Gene-Expression Miner dataset, *SLC7A11* mRNA expression was significantly associated with higher tumor grade, lymph node negative tumors, and high NPI (all *p* ≤ .0005; Supplementary Figure S3a-c).

**Table 1.** SLC7A11 copy number variation in the METABRIC breast cancer cohort and their association with clinicopathological parameters and breast cancer subtypes.

	Gain				Loss			
Parameters	No Number (%)	Yes Number (%)	χ2 (p-value)	Adjusted p-value	No Number (%)	Yes Number (%)	χ2 (p-value)	Adjusted p-value
***SLC7A11*****copy number**								
**Tumor size**								
<2.0 cm	616 (99)	6 (1)	.551	1.96	599 (96.3)	23 (3.7)	.595	1.89
≥2.0 cm	1314 (98.7)	17 (1.3)			1288 (96.8)	43 (3.2)		
**Tumor grade**								
1	167 (98.2)	3 (1.8)	.339	1.80	168 (98.8)	2 (1.2)	.092	.81
2	764 (99.2)	6 (0.8)			747 (97.0)	23 (3.0)		
3	938 (98.5)	14 (1.5)			912 (95.8)	40 (4.2)		
**Lymph Node Stage**								
1	1026 (99.1)	9 (0.9)	.393	1.86	1000 (96.6)	35 (3.4)	.112	.88
2	612 (98.4)	10 (1.6)			596 (95.8)	26 (4.2)		
3	312 (98.7)	4 (1.3)			311 (98.4)	5 (1.6)		
**Nottingham Prognostic Index**								
Good	676 (99.4)	4 (0.6)	.226	1.70	665 (97.8)	15 (2.2)	.015	.13
Moderate	1085 (98.5)	16 (1.5)			1053 (95.6)	48 (4.4)		
Poor	196 (98.5)	3 (1.5)			196 (98.5)	3 (1.5)		
**Histological type**								
Ductal	1638 (98.9)	19 (1.1)	.000014	.0002	1597 (96.4)	60 (3.6)	.637	2.36
Lobular	235 (99.6)	1 (0.4)			231 (97.9)	5 (2.1)		
Medullary	31 (96.9)	1 (3.1)			31 (96.9)	1 (3.1)		
Miscellaneous	36 (97.3)	1 (2.7)			37 (100)	0 (0.0)		
Special type	2 (66.7)	1 (33.3)			3 (100)	0 (0.0)		
**PAM50 subtype**								
Luminal A	712 (99.2)	6 (0.8)	.278	1.76	696 (96.9)	22 (3.1)	.084	.80
Luminal B	481 (98.6)	7 (1.4)			470 (96.3)	18 (3.7)		
HER2	236 (98.3)	4 (1.7)			235 (97.9)	5 (2.1)		
Basal	323 (98.2)	6 (1.8)			311 (94.5)	18 (5.5)		
Normal-like	199 (100)	0 (0.0)			196 (98.5)	3 (1.5)		
**METABRIC Integrative Cluster Membership**								
1	135 (97.1)	4 (2.9)	.07	.77	134 (96.4)	5 (3.6)	.033	.36
2	70 (92.7)	2 (7.3)			67 (93.1)	5 (6.9)		
3	289 (99.7)	1 (0.3)			282 (97.2)	8 (2.8)		
4	340 (99.1)	3 (0.9)			338 (98.5)	5 (1.5)		
5	187 (98.4)	3 (1.6)			185 (97.4)	5 (1.5)		
6	85 (100)	0 (0.0)			81 (95.3)	4 (4.7)		
7	186 (97.9)	4 (2.1)			185 (97.4)	5 (1.5)		
8	299 (100)	0 (0.0)			290 (97.0)	9 (3.0)		
9	145 (99.3)	1 (0.7)			142 (97.3)	4 (2.7)		
10	221 (97.8)	5 (1.2)			210 (92.9)	16 (7.1)		
**ER**								
Positive	1492 (99.1)	14 (0.9)	.086	.80	1460 (96.9)	46 (3.1)	.218	1.32
Negative	465 (98.1)	9 (1.9)			454 (95.8)	20 (4.2)		
**PR**								
Positive	1031 (99.1)	9 (0.9)	.196	1.65	1008 (96.9)	32 (3.1)	.504	1.80
Negative	926 (98.5)	14 (1.5)			906 (96.4)	34 (3.6)		
**HER2**								
Positive	239 (96.8)	8 (3.2)	.001	.01	242 (98.0)	5 (2.0)	.221	1.47
Negative	1718 (99.1)	15 (0.9)			1672 (96.5)	61 (3.5)		
**Triple Negative**								
Positive	315 (98.4)	5 (1.6)	.465	1.94	303 (94.7)	17 (5.3)	.031	.33
Negative	1642 (98.9)	18 (1.1)			1611 (97.0)	49 (3.0)		

*P*-values in bold denotes statistically significant.

At the protein level, high SLC7A11 was significantly associated with both high and low tumor grade (*p* = .02, Table 2) which was reflected in its separate components where it was associated with high mitotic count (*p* = .02, Table 2) and high pleomorphism (*p* = .002, Table 2) but in contrast also showed association with tubule formation (*p* = .007; Table 2). High SLC7A1l was further significantly associated with special and tubular histological types (*p* = .005; Table 2). There was no association between SLC7A11 with tumor size, nodal stage, or NPI.

**Table 2.** Clinicopathological associations of SLC7A11 protein expression in Nottingham BC cohort.

Parameters	Low n (%)	High n (%)	χ2 (p-value)	Adjusted P value
**SLC7A11 protein**				
**Tumor size**				
<2.0 cm	644 (60.5)	420 (39.5)	0.008 (0.92)	1.00
≥2.0 cm	552 (60.3)	363 (39.7)		
**Tumor Grade**				
1	156 (56.3)	121 (43.7)	8.66 (0.01)	**0.02**
2	473 (64.5)	260 (35.5)		
3	566 (58.5)	402 (41.5)		
**Mitosis**				
1	492 (63.0)	289 (37.0)	8.45 (0.01)	**0.02**
2	248 (62.8)	147 (37.2)		
3	432 (56.3)	335 (43.7)		
**Pleomorphism**				
1	19 (70.4)	8 (29.6)	16.56 (0.0002)	**0.002**
2	400 (66.7)	200 (33.3)		
3	754 (57.2)	564 (42.8)		
**Tubule formation**				
1	55 (49.1)	57 (50.9)	12.57 (0.002)	**0.007**
2	346 (56.9)	262 (43.1)		
3	772 (63.0)	453 (37.0)		
**Lymph Node Stage**				
1	744 (62.4)	449 (37.6)	4.89 (0.08)	0.13
2	342 (57.3)	255 (42.7)		
3	108 (57.8)	79 (42.2)		
**NPI**				
Good	365 (62.4)	220 (37.6)	1.71 (0.42)	0.50
Moderate	628 (60.0)	419 (40.0)		
Poor	201 (58.3)	144 (41.7)		
**Histological type**				
Ductal (including mixed)	1027 (59.4)	702 (40.6)	21.09 (0.001)	**0.005**
Lobular	118 (76.1)	37 (23.9)		
Medullary	13 (54.2)	11 (45.8)		
Miscellaneous	7 (77.8)	2 (22.2)		
Special type	30 (50.0)	30 (50.0)		
Tubular	1 (50.0)	1 (50.0)		

*P* values in bold means statistically significant.

### Association of SLC7A11 expression with BC subtypes

While *SLC7A11* CN gain was mainly associated with HER2+ cases (*p* = .01, Table 1), high levels of *SLC7A11* mRNA expression were observed in basal-like tumors (*p* < .001, Figure 2f) and METABRIC Integrative Clusters 2 (Luminal B) and 10 which embraces TNBC (*p* = .02, Figure 2d). There was no association between *SLC7A11* mRNA with ER, PR, or HER2. Within the Breast Cancer Gene-Expression Miner dataset, high *SLC7A11* was significantly associated with ER-, PR-, and HER2- BC (all *p* < .01; Supplementary Figure S3f-h). Both basal-like and luminal B tumors displayed high *SLC7A11* mRNA expression (*p* < .0001; Supplementary Figure S3d-e). High SLC7A11 protein was significantly associated with ER- tumors (*p* = .0006, Table 3). There was a higher expression of SLC7A11 protein in TNBC (*p* ≤ .04, Table 3).

**Table 3.** Association of SLC7A11 protein expression with breast cancer subtypes in the Nottingham BC cohort.

	SLC7A11 protein			
Subtypes	Low n (%)	High n (%)	χ2 (p-value)	Adjusted p-value
**ER**				
Negative	222 (52.5)	201 (47.5)	14.01 (0.0001)	**0.0006**
Positive	971 (62.5)	582 (37.5)		
**PR**				
Negative	461 (58.2)	331 (41.8)	2.73 (0.09)	0.27
Positive	716 (61.9)	440 (38.1)		
**HER2**				
Negative	1022 (61.0)	654 (39.0)	0.94 (0.33)	0.66
Positive	158 (57.9)	115 (42.1)		
**Triple Negative**				
No	1031 (61.9)	635 (38.1)	9.69	**0.008**
Yes	157 (52.3)	143 (47.7)	(0.002)	
**IHC subtypes**				
ER+/HER2- Low Proliferation	634 (63.2)	369 (36.8)	12.97 (0.01)	**0.04**
ER+/HER2- High Proliferation	201 (60.0)	134 (40.0)		
Triple Negative	159 (52.6)	143 (47.4)		
HER2+	118 (56.5)	91 (43.5)		

*P*-values in bold means statistically significant.

### *Association of* SLC7A11 *expression with immune infiltration*

Using the TIMER dataset, *SLC7A11* gene expression was positively associated, albeit weakly, in breast tumors with neutrophil (*p* = 2.0×10^−24^) and macrophage (*p* = 4.5×10^−6^) infiltration (Table 4). HER2+, luminal A and luminal B tumors also showed a weak to moderate correlation between *SLC7A11* mRNA with neutrophil and macrophage infiltration (*p* ≤ .009, Table 4). A weak negative correlation between *SLC7A11* mRNA and CD4+ immune cells was observed in HER2+, luminal A and luminal B tumors (*p* ≤ .037, Table 4). *SLC7A11* CN gain or loss were not associated with immune cell infiltrates in BC or any subtype (data not shown).

**Table 4.** SLC7A11 gene expression and association with immune infiltrates using timer.

Immune cell type	All cases (*n* = 1100)		Basal (*n* = 191)		HER2+. (*n* = 82)		Luminal A (*n* = 568)		Luminal B (*n* = 219)	
	Correlation co-efficient	p-value	Correlation co-efficient	p-value	Correlation co-efficient	p-value	Correlation co-efficient	p-value	Correlation co-efficient	p-value
*SLC7A11* mRNA										
CD8+	0.021	0.505	0.059	0.442	0.144	0.227	0.111	**0.012**	0.111	0.125
CD4+	−0.059	0.064	0.044	0.565	−0.353	**0.002**	−0.156	**3.9×10^−4^**	−0.151	**0.037**
B cell	−0.071	**0.026**	−0.138	0.070	−0.122	0.307	−0.036	0.415	−0.086	0.235
Neutrophil	0.316	**2.0×10^−24^**	0.247	**0.001**	0.307	**0.009**	0.260	**1.9×10^−9^**	0.197	**0.006**
Macrophage	0.145	**4.5×10**^−**6**^	0.014	0.858	0.463	**4.1×10**^−**5**^	0.196	**7.1×10^−6^**	0.275	**0.0001**

### SLC7A11 expression and other associated genes

The METABRIC dataset was used to investigate the correlation between *SLC7A11* mRNA and the expression of other associated genes (Table 5). The genes were selected based on other publications, being either regulatory genes or others that share SLC7A11 biological function.^30–33^ There was a positive correlation between *SLC7A11* mRNA expression and the expression of the regulatory gene, *ATF4* (*p* = .004, Table 5). *SLC7A11* was also positively correlated with the ancillary genes, *SLC3A2* and *CD44* (all *p* ≤ .01, Table 5). While the former was associated within all molecular subtypes (all *p* ≤ .03, Table 5) except HER2+, correlation with *CD44* was only observed within luminal B tumors (*p* = .03, Table 5). Whilst there was a positive correlation observed between *SLC7A11* with the high glutamine affinity solute carriers *SLC1A5* and *SLC7A5* (all *p* ≤ .001, Table 5). There were no significant associations with other glutamine affinity solute carriers *SLC7A8* or *SLC38A2*. Likewise, there was no association between *SLC7A11* and the glutamate transporters *SLC1A3*, *SLC1A6* and *SLC1A7* or the cystine/glutamate antiporter *SLC1A1*. There was, however, a positive association between *SLC7A11* and *SLC1A2* (*p* = .04, Table 5) which lost its significance when the different BC subtypes were examined separately. The association of SLC7A11 protein expression with other associated proteins was also examined (Table 6). At the protein level, SLC7A11 was significantly expressed with breast tumors that expressed high MYC, Ki67, and Glutaminase (GLS; all *p* ≤ .01, Table 6). There was no significant association between SLC7A11 and Glutamate Dehydrogenase (GLUD1) (*p* > .05, Table 6), the enzyme which degrades glutamate to α-ketoglutarate. Nonetheless, a positive association was observed with SLC7A11 and SLC1A5, SLC7A5, SLC7A8, SLC3A2 and SLC38A2 (all *p* ≤ .04, Table 6). Moreover, SLC7A11 was highly expressed in BCs which express high p53 protein (*p* = .01, Table 6).

**Table 5.** Correlation of *SLC7A11* mRNA expression with the expression of other related genes in the METABRIC cohort.

	*SLC7A11* mRNA											
	All cases (*n* = 1,980)		Luminal A (*n* = 368)			Luminal B (*n* = 367)			HER2+ (*n* = 110)		Triple negative (*n* = 150)	
	Coefficient Correlation (p-value)	Adjusted p-value	Coefficient Correlation (p-value)		Adjusted p-value	Coefficient Correlation (p-value)		Adjusted p-value	Coefficient Correlation (p-value)	Adjusted p-value	Coefficient Correlation (p-value)	Adjusted p-value
**Regulatory and other associated genes**												
*MYC*	0.02 (0.38)	2.48	0.02 (0.43)		2.64	0.02 (0.60)		1.64	0.01 (0.78)	5.20	−0.04 (0.38)	3.44
*ETS-1*	−0.05 (0.01)	0.12	−0.08 (0.02)		0.32	−0.06 (0.17)		1.30	−0.08 (0.17)	2.76	0.02 (0.59)	3.72
*NRF2*	−0.02 (0.25)	1.75	−0.02 (0.44)		2.73	−0.05 (0.20)		1.40	−0.02 (0.70)	4.80	0.06 (0.26)	2.68
*ATF4*	0.08 (0.0003)	0.004	0.07 (0.05)		0.75	0.06 (0.17)		1.36	0.06 (0.29)	2.88	0.08 (0.13)	1.96
*CD44*	0.07 (0.001)	**0.01**	0.007 (0.85)		3.87	0.13 (0.002)		**0.03**	0.19 (0.003)	0.05	0.06 (0.25)	2.08
**Enzymes involved in glutamine metabolism**												
*GLS*	0.01 (0.48)	2.50	−0.02 (0.58)	3.30		0.06 (0.13)	1.26		0.06 (0.32)	2.89	0.04 (0.43)	3.50
*GLUL*	0.03 (0.14)	1.44	0.02 (0.47)	2.73		0.01 (0.82)	3.00		0.04 (0.52)	4.64	0.15 (0.006)	0.10
*GLUD1*	0.02 (0.31)	1.75	0.01 (0.64)	3.52		0.11 (0.01)	0.15		0.02 (0.69)	4.76	−0.06 (0.25)	2.43
**Glutamine transporters**												
*SLC3A2*	0.13 (1.1×10–9)	<0.0001	0.11 (0.002)	0.03		0.14 (0.001)	0.01		0.05 (0.40)	4.26	0.17 (0.002)	0.03
*SLC1A5*	0.13 (2.1×10–9)	<0.0001	0.15 (0.00004)	0.0008		0.13 (0.003)	0.04		−0.02 (0.71)	4.84	0.11 (0.05)	0.85
*SLC7A5*	0.08 (0.00008)	0.001	0.03 (0.37)	2.64		0.08 (0.06)	0.77		.09 (0.16)	4.97	0.05 (0.36)	3.42
*SLC7A8*	−0.007 (0.75)	2.90	−0.04 (0.22)	2.32		0.05 (0.21)	1.53		0.04 (0.44)	4.42	0.05 (0.32)	2.95
*SLC38A2*	−0.03 (0.15)	1.50	−0.04 (0.21)	1.70		−0.02 (0.60)	1.64		−0.01 (0.83)	5.60	0.02 (0.67)	3.75
**Cystine/glutamate transporters**												
*SLC1A1*	0.06 (0.006)	0.07	0.09 (0.01)	0.17		0.10 (0.02)	0.28		0.006 (0.92)	6.10	0.01 (0.81)	3.80
*SLC1A2*	0.06 (0.003)	0.04	0.04 (0.21)	1.92		0.08 (0.07)	0.78		0.06 (0.34)	3.32	0.03 (0.53)	3.52
**Glutamate transporters**												
*SLC1A3*	−0.02 (0.35)	2.10	−0.02 (0.55)	2.80		−0.11 (0.01)	0.16		−0.06 (0.34)	3.90	0.05 (0.32)	3.38
*SLC1A6*	0.02 (0.35)	2.45	0.02 (0.56)	3.29		0.08 (0.07)	0.84		−0.02 (0.71)	4.97	−0.03 (0.58)	3.60
*SLC1A7*	−0.02 (0.35)	2.45	−0.04 (0.24)	2.44		−0.01 (0.73)	2.40		0.004 (0.95)	6.21	−0.01 (0.98)	3.84

*P* values in bold means statistically significant.

**Table 6.** Association of SLC7A11 protein expression and other associated proteins in the Nottingham BC cohort.

	SLC7A11 protein			
Protein	Low n (%)	High n (%)	χ2 (p-value)	Adjusted p-value
**c-MYC**				
Negative	328 (58.7)	231 (41.3)	21.28 (0.000004)	**<0.0001**
Positive	22 (30.1)	51 (69.9)		
**Ki67**				
Negative	122 (64.6)	67 (35.4)	9.67 (0.002)	**0.01**
Positive	219 (51.0)	210 (49.0)		
**GLS**				
Negative	252 (63.8)	143 (36.2)	27.11 (1.9×10^−7^)	**<0.0001**
Positive	123 (43.6)	159 (56.4)		
**GLUD1**				
Negative	212 (57.3)	158 (42.7)	0.73 (0.39)	0.78
Positive	185 (54.1)	157 (45.9)		
**SLC1A5**				
Negative	430 (68.6)	197 (31.4)	27.80 (1.3×10^−7^)	**<0.0001**
Positive	686 (55.9)	541 (44.1)		
**SLC7A5**				
Negative	942 (62.0)	577 (38.0)	13.22 (0.0002)	**0.001**
Positive	189 (51.6)	177 (48.4)		
**SLC3A2**				
Negative	778 (62.1)	474 (37.9)	9.25 (0.002)	**0.01**
Positive	261 (54.1)	221 (45.9)		
**SLC38A2**				
Negative	765 (61.9)	470 (38.1)	6.25 (0.01)	**0.04**
Positive	62 (50.4)	61 (49.6)		
**SLC7A8**				
Negative	744 (64.5)	410 (35.5)	11.20 (0.001)	**0.005**
Positive	70 (50.0)	70 (50.0)		
**p53**				
Negative	770 (62.7)	458 (37.3)	9.57	**0.01**
Positive	350 (55.3)	283 (44.7)	(0.002)	

*P* values in bold means statistically significant.

### Association of SLC7A11 and patient outcome

**Figure 3.**
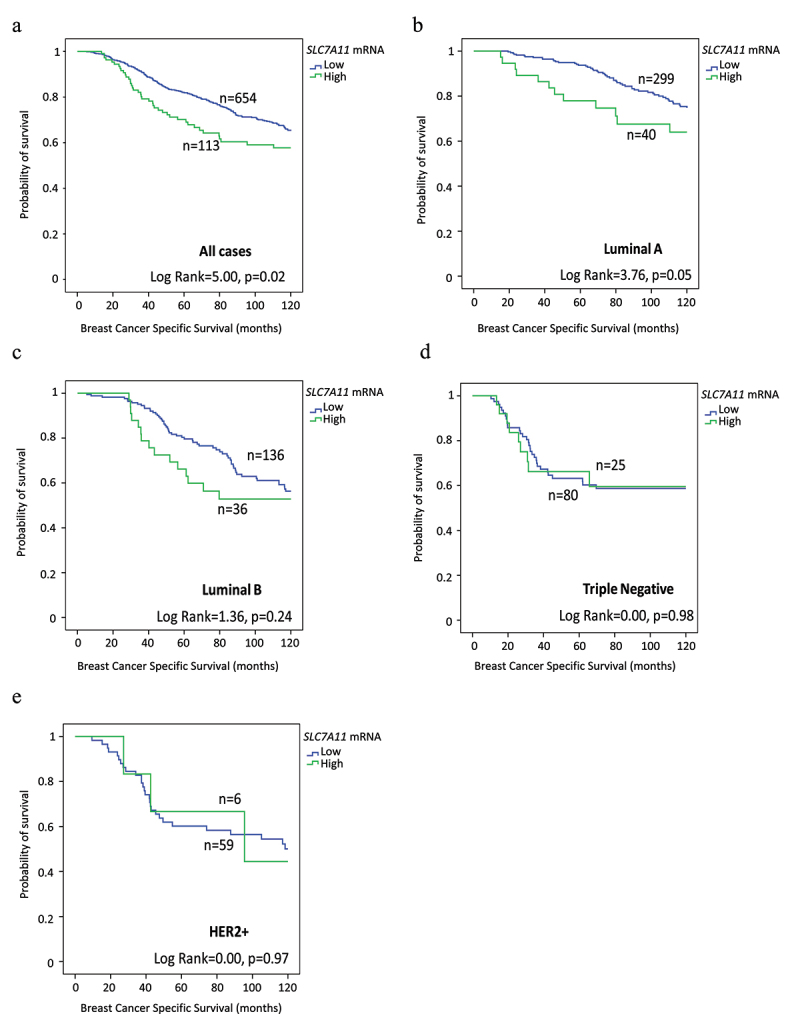
*SLC7A11* mRNA expression and its association in breast cancer biological subtypes with patient breast cancer specific survival in the METABRIC cohort: a) *SLC7A11* in all cases, b) *SLC7A11* in luminal A, c) *SLC7A11* in luminal B, d) *SLC7A11* vs triple Negative, and e) *SLC7A11* in HER2+ breast cancer.

**Figure 4.**
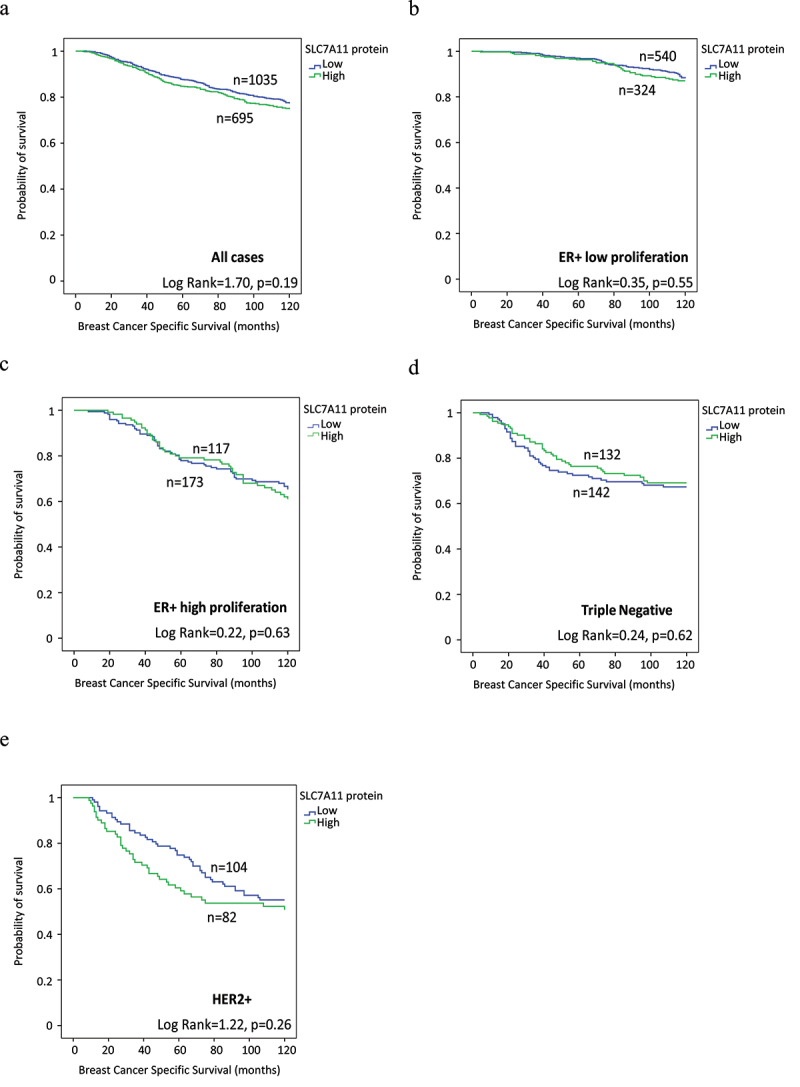
SLC7A11 protein expression and its association with patient breast cancer specific survival (BCSS) in the Nottingham cohort: a) SLC7A11 in all cases, b) SLC7A11 in ER+ low proliferative, c) SLC7A11 in ER+ high proliferative, d) SLC7A11 in triple Negative, and e) SLC7A11 in HER2+ breast cancer.

Survival analysis of high *SLC7A11* mRNA was significantly associated with shorter BCSS (*p* = .02, Figure 3a). Multivariate analysis showed that *SLC7A11* mRNA was a predictive of poor survival independent of tumor grade, tumor size, and lymph node stage (*p* = .008, Table 7) and the result remained significant within the luminal A tumors (*p* = .02, Table 7). The relationships between *SLC7A11* mRNA expression and patient outcome were verified using Breast Cancer Gene-Expression Miner (Supplementary Figure S4). High *SLC7A11* mRNA expression showed association with shorter overall survival (OS) in all cases (*p* = .0004, Supplementary Figure S4a) and in HER2+ cases (*p* = .02, Supplementary Figure S4e). In contrast, SLC7A11 protein was not associated with patient outcome, in terms of BCSS and DMFS either in the whole cohort or when the different BC subtypes were examined (Figure 4). High *SLC7A11* mRNA expression showed association with shorter OS (*p* = 3.6×10–5, Supplementary Figure S5a) in the KM plotter dataset whereas no significance was found in terms of SLC7A11 protein expression (Supplementary Figure S5b). DNA methylation of both cg21877274 and cg24869834 CpG sites on SLC7A11 correlated with poorer patient survival rates (*p* = .0014 and *p* = .0074 respectively; Supplementary Figure S2b,c).

**Table 7.** SLC7A11 mRNA expression and patient outcome in BC molecular subtypes using the METABRIC cohort.

	*SLC7A11* mRNA									
	All cases		Luminal A		Luminal B		HER2+		Triple negative	
*Parameter*	Hazard ratio (95% CI)	p-value	Hazard ratio (95% CI)	p-value	Hazard ratio (95% CI)	p-value	Hazard ratio (95% CI)	p-value	Hazard ratio (95% CI)	p-value
*SLC7A11*	1.57 (1.12–2.21)	**0.008**	2.08 (1.11–3.84)	**0.02**	1.29 (0.73–2.30)	0.37	1.11 (0.52–2.36)	0.77	1.26 (0.36–4.38)	0.71
Nodal stage	1.71 (1.43–2.04)	**2.1×10^−9^**	1.31 (0.93–1.84)	0.12	1.82 (1.34–2.48)	**0.0001**	1.89 (1.19–3.01)	**0.007**	2.19 (1.31–3.65)	**0.003**
Tumor size	1.51 (1.07–2.14)	**0.01**	2.03 (1.11–3.73)	**0.02**	1.56 (0.80–3.04)	0.19	0.77 (0.37–1.58)	0.48	2.47 (0.72–8.46)	0.14
Tumor grade	1.45 (1.14–1.86)	**0.003**	1.23 (0.81–1.87)	0.33	0.96 (0.62–1.49)	0.88	2.95 (0.39–21.84)	0.28	0.71 (0.32–1.58)	0.41

*P* values in bold means statistically significant.

## Discussion

BC is a biologically heterogeneous disease^34^ with various molecular subtypes that influences its metabolic programming and nutritional needs for its growth and proliferation, which can ultimately affect patient survival. Thus, understanding the metabolic profiling along with their biological characteristics could help to identify potential therapeutic targets and prognostic markers. The present study explored SLC7A11 expression in BC by assessing its genomic, transcriptomic, and proteomic levels for the first time in several large breast cancer cohorts. It revealed that SLC7A11 could potentially play a significant role in BC subtypes influencing the patient outcome.

In this study, we show *SLC7A11* CN loss was more common than CN gain which was generally associated with a small number of HER2+ cases. However, there was no association between *SLC7A11* CNV and mRNA expression, suggesting that CN gain does not contribute to *SLC7A11* transcription or SLC7A11 translation. *SLC7A11* mRNA and SLC7A11 protein expression were primarily associated with poor clinicopathological parameters and *SLC7A11* DNA methylated predicted poor patient survival. Further analysis revealed that SLC7A11 was positively correlated with other amino acid transporters and enzymes associated with glutamine metabolism, implying a coordinated role in metabolic regulation. Additionally, *SLC7A11* gene expression was positively associated with neutrophil and macrophage infiltration, suggesting a potential link between SLC7A11 and tumor immunity.

While focusing on BC subtypes, the poor prognostic ER negative and TNBC showed high *SLC7A11* mRNA and SLC7A11 protein expression. This relation was supported by a previous study where Timmerman et al^26^ reported that SLC7A11 is significantly expressed in TNBC. TNBC tend to act as a glutamine auxotroph, which correlates SCL7A11 and cystine consumption. This potentially indicates that TNBC with high SLC7A11 expression can show an aggressive BC advancement. TNBC currently as only chemotherapy as its treatment option due to the absence of growth in response to hormones and SLC7A11 is found to modulate chemotherapy or vice versa.^35,36^ Cancer cells are rendered more resistant to chemotherapy if SLC7A11 is overexpressed.^37^ This supports the fact that SLC7A11 can represent itself as a potential target to observe it as a treatment response factor in TNBC and such suggestions were backed by earlier studies.^25,35,38^

Regarding exploring the additional genes associated with *SLC7A11*, transcription factor *ATF4* was significantly associated with *SLC7A11* mRNA. This transcription factor is thought to foster SLC7A11 expression and upregulating *SLC7A11* transcription.^39–41^ AFT4 regulates redox homeostasis and amino acid metabolism.^38^ As a part of redox homeostasis in the cancer cell, SLC7A11 presence contributes to increasing glutathione, which later promotes metabolic reprogramming. This supports the notion that under stress condition; proteasome inhibition or glucose starvation, SLC7A11 requires ATF4 for its expression.^40^ This relation can be targeted for SLC7A11 reduction supported by previous studies showed AFT4 inhibition reduces SLC7A11 expression.^39,41–43^ Hereby, SLC7A11 is also influenced by CD44, the adhesion molecule, which stabilizes SLC7A11 which subsequently promotes the uptake of cystine for glutathione synthesis,^33^ which is further supported by the fact that CD44 deficiency can result into intracellular glutathione depletion^43^ resulting in ROS induction, inhibiting tumor formation and inducing ferroptosis. From our study, *SLC7A11* mRNA shows a strong relation with CD44, distinctively within luminal B tumors, and this reflects previous evidence.^44–46^

This study further investigated the association of SLC7A11 expression with other glutamine transporters. SLC7A11 was strongly associated with SLC3A2, SLC1A5, SLC7A5, and SLC38A2, which reinforces that amino acid transporter activity is altered to meet the altered environment in BC. SLC3A2 is required for SLC7A11 for its optimum activation, where SLC7A11 heterodimerises with SLC3A2 via a disulfide bridge. Efficient exchange of glutamate requires both subunits where the transport activity which is primarily maintained by SLC7A11, highly specific for cystine and glutamate, whereas SLC3A2 which mainly acts as a chaperone protein to regulate the trafficking function of SLC7A11.^47–49^ SLC3A2 absence also brings a substantial decrease of SLC7A11 activity indicating a crucial role for SLC7A11 stability.^50,51^

Our study also showed SLC7A11 protein was strongly associated with GLS expression, the key enzyme for glutamine catabolism in line with an earlier study.^52^ GLS activity is paused by the presence of sufficient intracellular glutamate but the high expression of SLC7A11, which decreases the cellular glutamate by exporting, potentially reactivate GLS activity through negative feedback contributing to cell nutrient supply, especially in TNBC.^53^ Thus, the combination of GLS and SLC7A11 inhibitors can offer a potential treatment approach for TNBC.

In BC, *MYC* and *TP53* genes are altered and have direct role on BC prognosis and treatment options.^54^ c-Myc has a direct role in glutamate metabolism^55^ and SLC7A11 is directly induced by c-Myc, or vice versa, and our results support this association. In addition, p53 protein (encoded by *TP53*) is strongly associated with SLC7A11^18^ and p53 mutations alter SLC7A11 expression.^51,56^ p53 also acts upon glutathione synthesis, which is mediated by SLC7A11, when tumor cells are serine starved, thus preserving cellular anti-oxidant capacity.^57^ p53 also negatively regulates SLC7A11 and as p53 is considered a pro-ferroptotic factor, it can increase the sensitivity to ferroptosis.^31^ Furthermore, high SLC7A11 expression was significantly associated with Ki67 reinforcing previous studies.^18,58^ Since Ki67 is a poor prognostic biomarker in invasive breast cancer,^59^ its positive association with SLC7A11 depicts the fact that subtype with overexpression of SLC7A11 can demonstrate aggressive disease progression.

*SLC7A11* mRNA was correlated with the infiltration levels of neutrophils and macrophages irrespective of biological subtype. Neutrophils, believed to promote metastasis in BC,^60^ can affect intracellular GSH levels and thus can influence SLC7A11 activity.^61^ Macrophages are thought to participate in ferroptosis^62^ and SLC7A11 could act as a connection between the two. High presence of these immune cells can potentially offer a synergistic impact for BC treatment focusing on SLC7A11.

Whilst high *SLC7A11* mRNA was an independent predictor of poor OS all cases and in luminal A tumors this was not translated into protein expression. This discrepancy can be attributed to transcriptional factors, miRNA, linc-RNA, DNA methylation, and other translational and post-translational modifications as well as protein stability, other co-existing metabolic pathway, cellular condition and coupling with other protein could change the protein activity so as of SLC7A11.^63–67^ Transcriptional process and mRNA decaying process can lead discordance between protein and mRNA expression. *SLC7A11* mRNA has a relation with *SLC3A2* and *CD44* which can also affect its protein stability, so these regulatory mechanisms require further investigation.

In conclusion, this study revealed a robust characterization of the glutamate/cysteine transporter SLC7A11 as an independent prognostic factor in BC. Several SLC and other factors associated with the glutamate transport axis were related to *SLC7A11* and their combined functionality gives opportunities to explore them as potential therapeutic targets. Further functionals studies on SLC7A11 in BC are therefore warranted.

## Material and methods

### SLC7A11 *genomic and transcriptomic expression*

*SLC7A11* CNV and mRNA expression were evaluated in a cohort of 1,980 BC tumors in the Molecular Taxonomy of Breast Cancer International Consortium (METABRIC).^68^ The METABRIC study provides genomic and transcriptional profiling data on BC using the Affymetrix SNP 6.0 and Illumina HT-12v3 platforms, respectively. In this cohort, patients enrolled as ER- and lymph node (LN) positive received adjuvant treatment while patients with ER+ and/or LN negative did not receive adjuvant chemotherapy. Dichotomization of *SLC7A11* mRNA expression was conducted using X-tile (version 3.6.1, Yale University, USA), based on the prediction of Breast Cancer Specific Survival (BCSS). Association of CNV including loss and gain and mRNA expression along with clinicopathological parameters and molecular subtypes were investigated. mRNA expression with other genes related to glutamine metabolism and patient’s survival were also investigated. To validate *SLC7A11* mRNA expression, Breast Cancer Gene Expression Miner v4.5 (bcgenex.centregauducheau.fr) incorporating The Cancer Genome Atlas (TCGA) and SCAN-B RNA sequencing data (*n* = 4,712) and Kaplan–Meier Plotter database (RNA-seq data for breast cancer) (*n* = 2976) were used as external validation datasets.

*SLC7A11* CNV (*n* = 1080) and mRNA (*n* = 1100) expression were also investigated in BC using TIMER (http://timer.cistrome.org/) to estimate the abundance of SLC7A11 in six subsets (B cells, CD4+ T cells, CD8+ T cells, macrophages, neutrophils, and dendritic cells).^69^ MethSurv (biit.cs.ut.ee/methsurv) was used to explore the DNA methylation of *SLC7A11* in the TCGA data according to patient survival.^70^ DNA methylation status was dichotomized using the ‘best’ option.

### Patients’ cohort for SLC7A11 protein expression

Immunohistochemistry (IHC) was conducted on a well-characterized BC series from patients at Nottingham City Hospital during 1987–2006 (*n* = 1,981). The patients were diagnosed with early-stage primary operable invasive BC and aged ≤70 years. Patient management was uniform and based on tumor characteristics including the Nottingham Prognostic Index (NPI) and hormone receptor status. Patients with NPI > 3.4 received tamoxifen if ER+ (± goserelin, in premenopausal patients). Conversely, classical cyclophosphamide, methotrexate and 5-fluorouracil (CMF) were used in ER-patients who were fit enough to receive chemotherapy. No patients received neoadjuvant therapy. Clinical history, tumor characteristics, information on therapy and outcomes were prospectively maintained. Outcome data including development and time to distant metastasis (DM) and BCSS. DM-free survival (DMFS) was defined as the time (in months) from the date of primary surgery to the appearance of DM. The BCSS was defined as the time (in months) from the date of primary surgery to the date of BC-related death. SLC7A11 protein expression was correlated with clinicopathological parameters, BC subtypes, other genes related to glutamine metabolism and patient’s survival. The Kaplan–Meier Plotter database (protein data for breast cancer) (*n* = 1064) was used as an external validation dataset.

### SLC7A11 antibody validation

SLC7A11 primary antibody specificity (Rabbit monoclonal, Ab37185, Abcam, UK) was determined using Western blotting in cell line lysates: HCC1500, ZR-751, MDA-MB-436, MCF7 and T47D (American Type Culture Collection; Rockville, MD, USA at a dilution of 1:2000. Donkey anti-rabbit (1:15,000, IRDye680 CW, 926–32213, LI-COR Bioscience) was used as a fluorescent secondary antibody. Mouse monoclonal anti-β-actin primary antibody (1:5,000, A5441, Sigma-Aldrich) with donkey anti-mouse fluorescent secondary (1:15,000, IRDye 800CW, 926–68072, LI-COR Bioscience) was used as a control. The Odyssey Fc machine (LI-COR Bioscience) was used to visualize blots showing specific bands at the predicted size of approximately 55 KDa (Supplementary Figure S1).

### Tissue microarrays and IHC

Tissue Microarrays (TMAs) were produced following standard techniques as described.^71^ Polymer-based IHC was performed on 4 µm thick section using the Novolink Polymer Detection System (Leica Biosystems, RE7150-K) according to the manufacturer’s instructions. Heat mediated antigen retrieval was carried out using citrate buffer at pH 6.0. SLC7A11 antibody was used at a 1:50 dilution for 1 hour at room temperature. Beta2-microglobulin at a concentration of 1:2,000 was used as a positive control.

### Assessment of SLC7A11 protein expression

Stained TMA sections were assessed using high resolution digital images (NanoZoomer, Hamamatsu Photonics, Welwyn Garden City, UK) at 20× magnification viewed using Philips Xplore software (Philips Digital Pathology Solutions, Amsterdam, Netherlands). Assessment of protein staining was based on a semi-quantitative H-Score which includes an assessment of both intensity of staining and percentage of stained cells.^72^ Adequate tumor burden was assessed ensuring that the TMA core contained more than 15% of invasive tumor (excluding DCIS). Intensity of the staining ranged from a scale of 0 to 3 (0 = no staining, 1= weak, 2 = moderate, 3 = strong) and the percentage of positively stained invasive BC cells was estimated subjectively. A final H-score of 0–300 was generated based on % of cells stained at each intensity. A second observer blindly scored 10% cores to assess scoring ability and concordance. There was high inter-observer concordance between the scorers (Kappa score = 0.64). Dichotomization of SLC7A11 protein expression was determined based on the prediction of BCSS using X-tile software.

Immunohistochemical staining and dichotomization of the other biomarkers included in this study were as per previous publications.^73–77^ ER and progesterone receptor (PR) positivity was defined as ≥ 1% staining. Immunoreactivity of HER2 in TMA cores was scored using standard HercepTest guidelines (Dako). Chromogenic in situ hybridization (CISH) was used to quantify HER2 gene amplification in borderline cases using the HER2 FISH pharmDx™ plus HER2 CISH pharmDx™ kit (Dako) and was assessed according to the American Society of Clinical Oncology guidelines. BC molecular subtypes were defined based on tumor IHC profile and the Elston-Ellis^78^ mitotic score as: ER+/HER2- low proliferation (mitotic score 1), ER+/HER2- high proliferation (mitotic score 2 and 3); HER2+ class: HER2+ regardless of ER status; triple negative: ER-, PR- and HER2-.^79^ Basal-like phenotype was defined as tumors expressing cytokeratin (Ck) 5/6, and/or Ck14 and/or Ck17.

### Statistical analysis

Statistical analysis was performed using SPSS 22 statistical software (SPSS Inc., Chicago, IL, USA). Chi-square test was performed to analyze categorical variables. For continuous variables, Pearson correlation coefficient was used to test correlation between two continuous normalized data, whereas the differences between three or more groups were assessed using one-way analysis of variance (ANOVA) with the post-hoc Tukey multiple comparison test. Survival analysis was done using Kaplan-Meier with Log Rank test for outcome. Cox’s proportional hazard method was implied to identify independent prognostic factors. P-values were adjusted using Bonferroni correction for multiple testing. Variables with p-values <0.05 were considered statistically significant.

## Supplementary Material

Supplemental Material

## Data Availability

The data that support the findings of this study are available from the corresponding author upon reasonable request.
