# Supplementary material for: The amino acid transporter SLC7A11 expression in breast cancer
Source: Cancer Biol Ther. 2023 Dec 10;25(1):2291855. doi: 10.1080/15384047.2023.2291855 (PMC10761065; doi:10.1080/15384047.2023.2291855)
Supplement: Supplemental Material [file KCBT_A_2291855_SM9537.docx]

**Supplementary Figure 1S:** SLC7A11 protein expression in breast cancer cell lines by Western blotting

**Supplementary Figure 2S:** *SLC7A11* DNA methylation in breast cancer using MethSurv: a) heat map, b-c) Kaplan-Meier survival analysis for patients with high and low *SLC7A11* DNA methylation levels at the cg21877274 and cg24869834 CpG sites.

**Supplementary Figure 3S:** *SLC7A11* gene expression and its association with clinicopathological parameters in the Breast Cancer Gene-Expression Miner RNA-sequencing dataset: a) *SLC7A11* and tumor grade (SBR), b) *SLC7A11* and nodal status, c) *SLC7A11* and Nottingham Prognostic Index (NPI), d) *SLC7A11* and histological type, e) *SLC7A11* and PAM50, f) *SLC7A11* and ER, g) *SLC7A11* and PR, and h) *SLC7A11* and HER2

**Supplementary Figure 4S:** *SLC7A11* gene expression and its association with patient overall survival in the Breast Cancer Gene-Expression Miner RNA-sequencing dataset a) *SLC7A11* in all cases, b) *SLC7A11* in Luminal A, c) *SLC7A11* in Luminal B, d) *SLC7A11* in basal-like, e) *SLC7A11* in HER2+, and f) *SLC7A11* in normal breast-like breast cancer

**Supplementary Figure 5S:** *SLC7A11* mRNA and SLC7A11 protein expression and its association with patient overall survival (OS) using the Kaplan-Meier Plotter a) *SLC7A11* mRNA in all cases, b) SLC7A11 protein in all cases


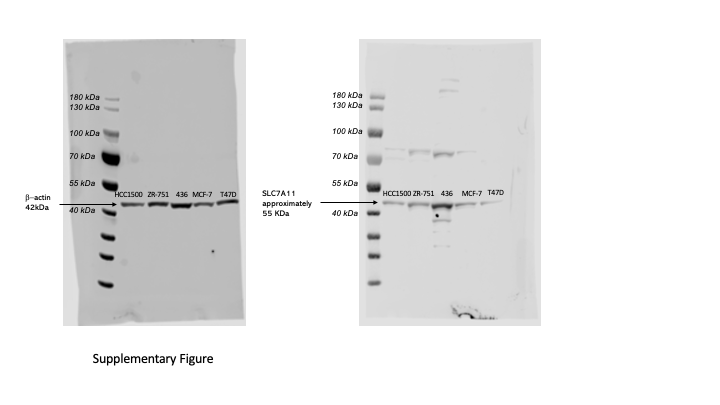


**Supplementary Figure 1S**

–

a


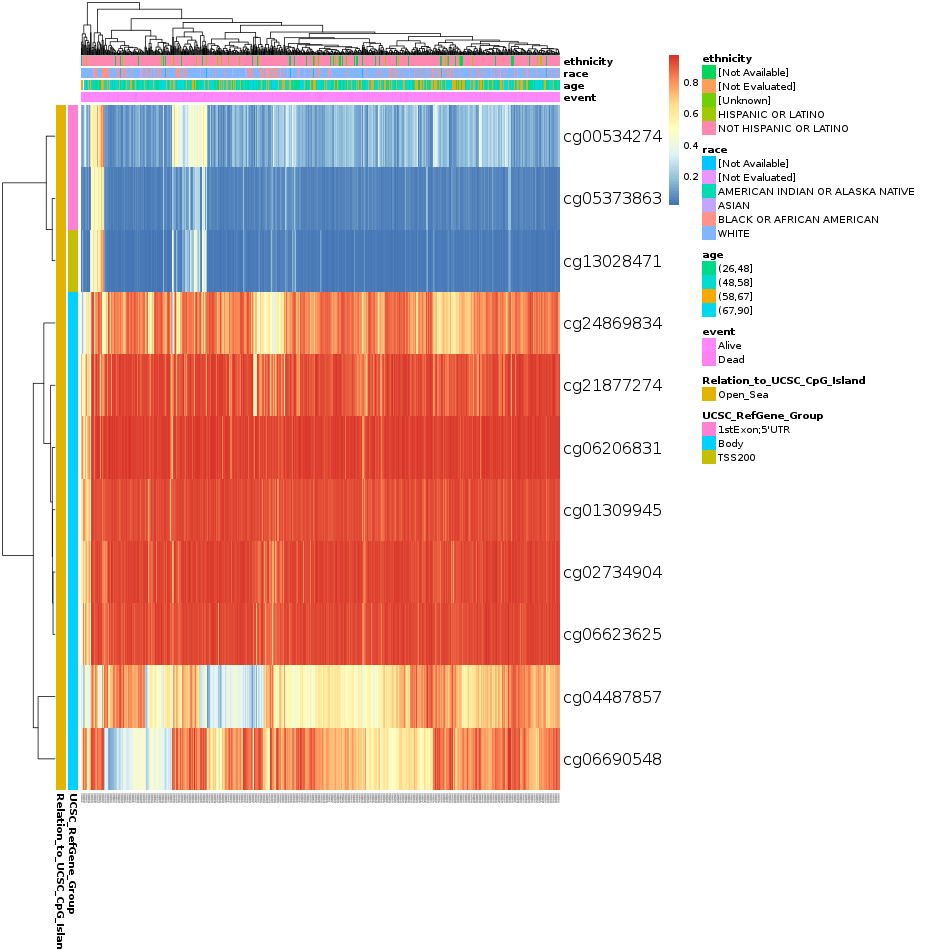

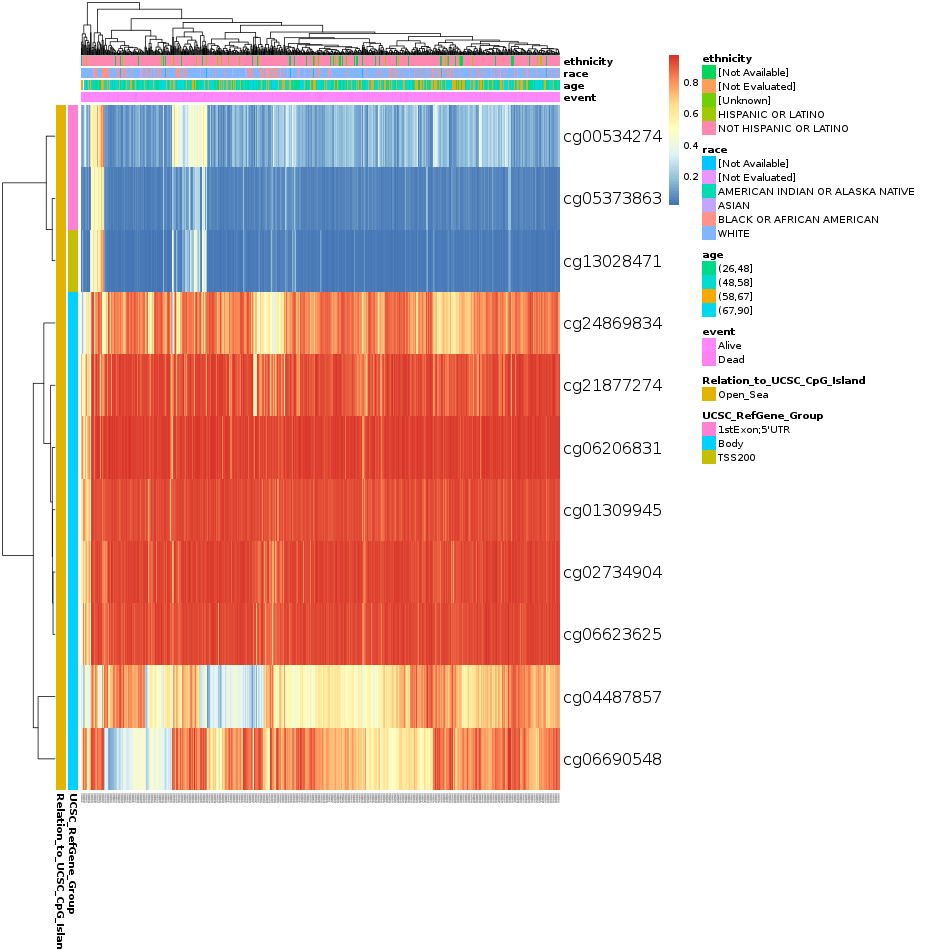

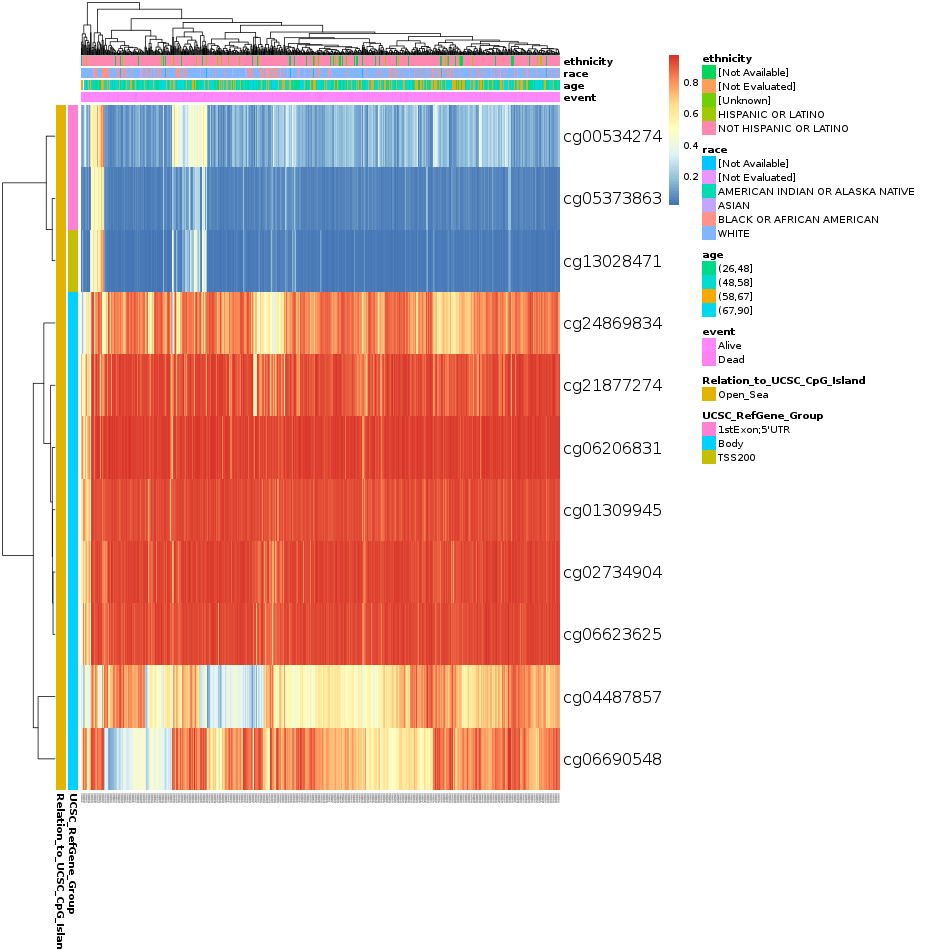


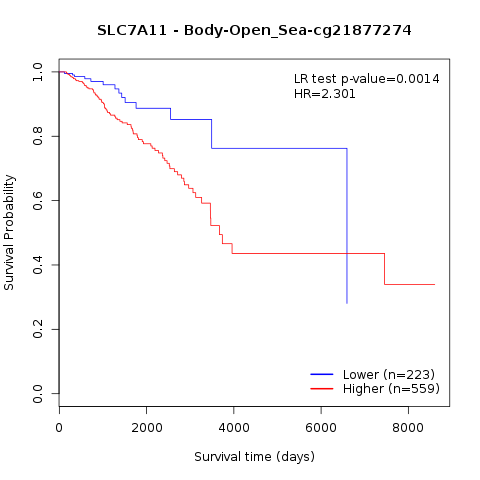

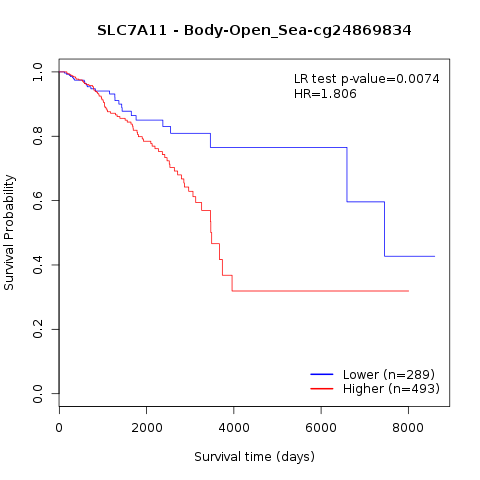

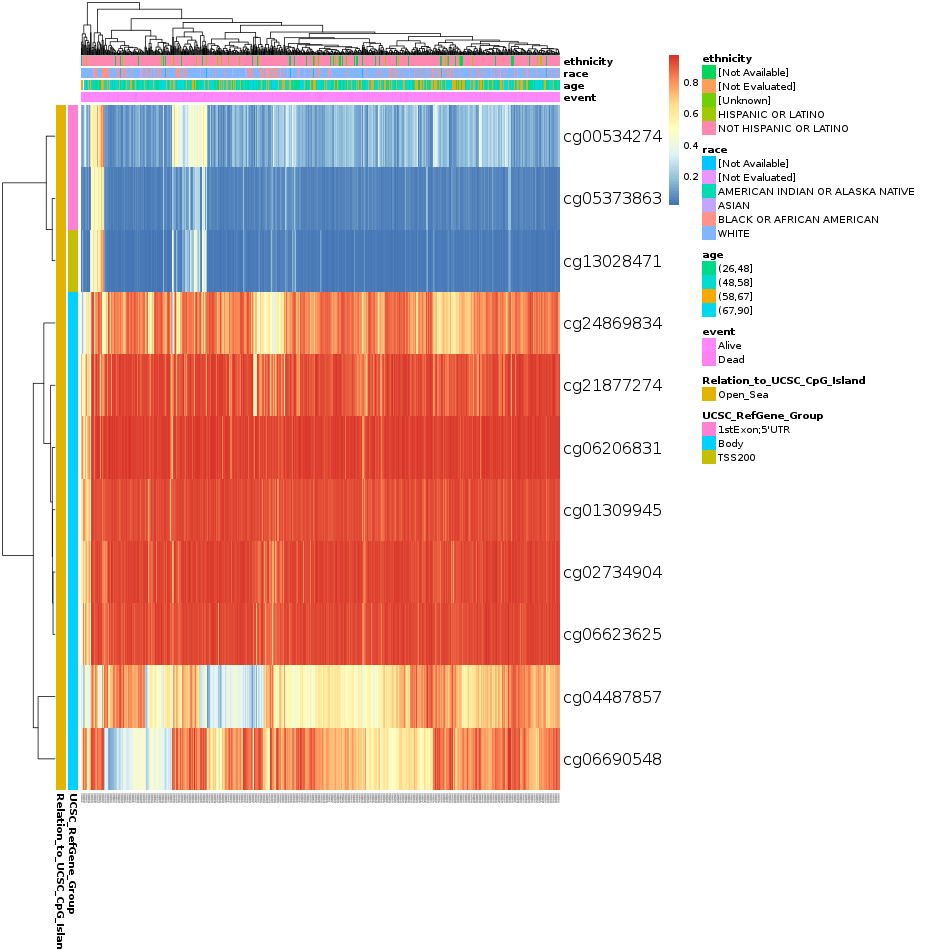


c

b

**Supplementary Figure 2S**


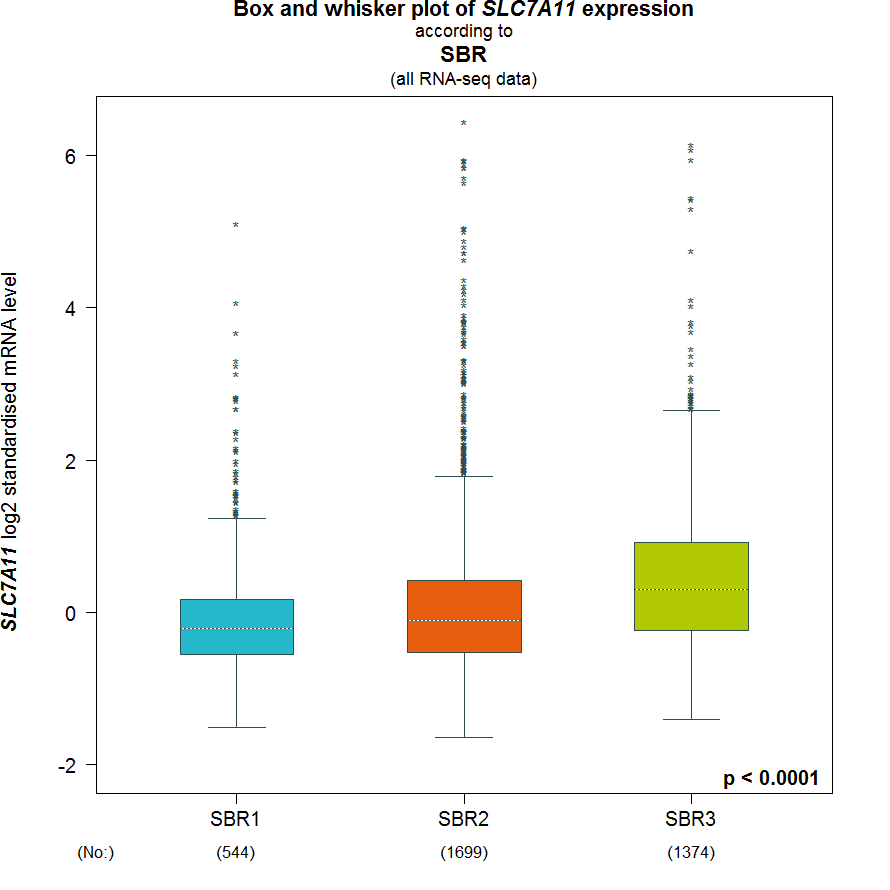

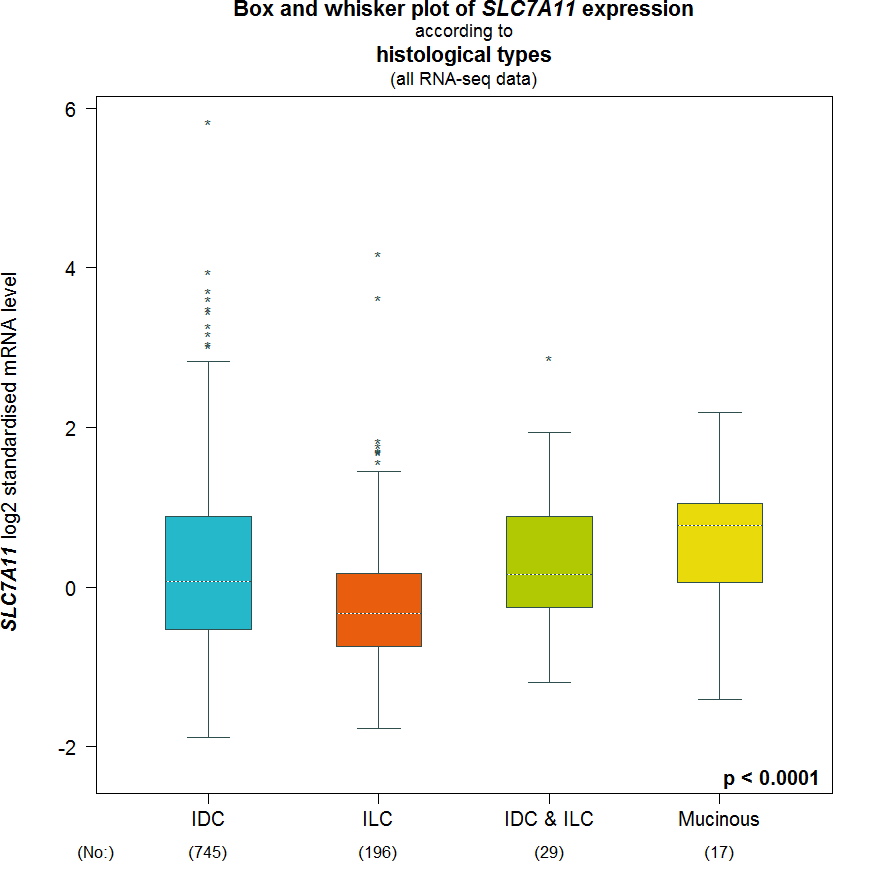

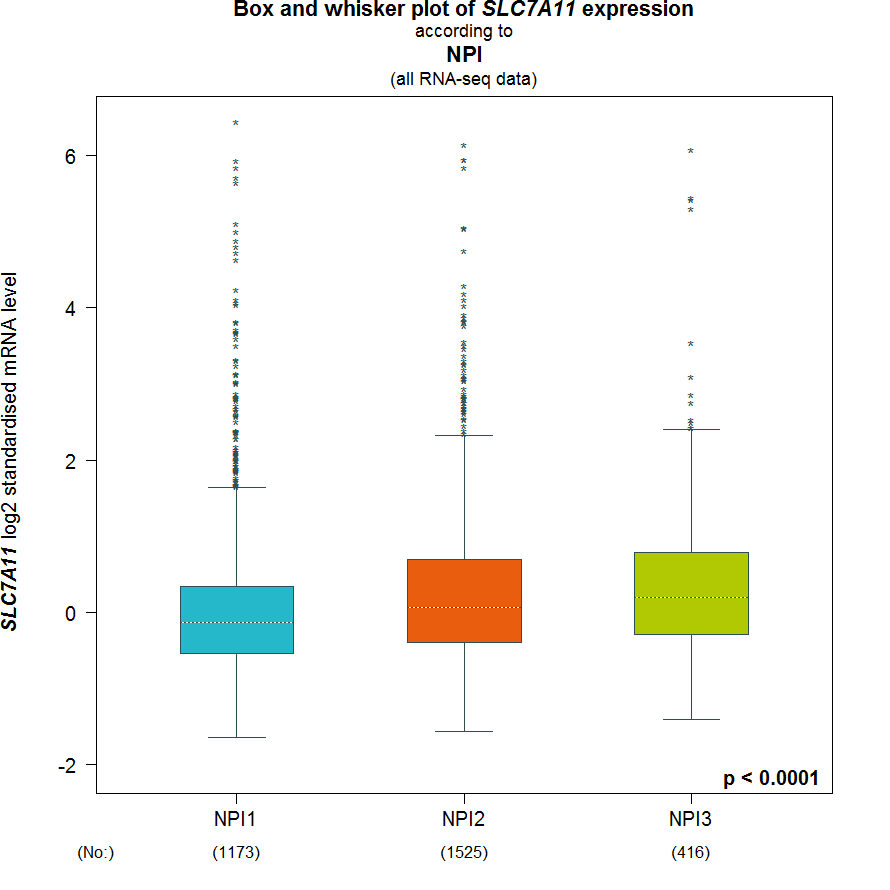

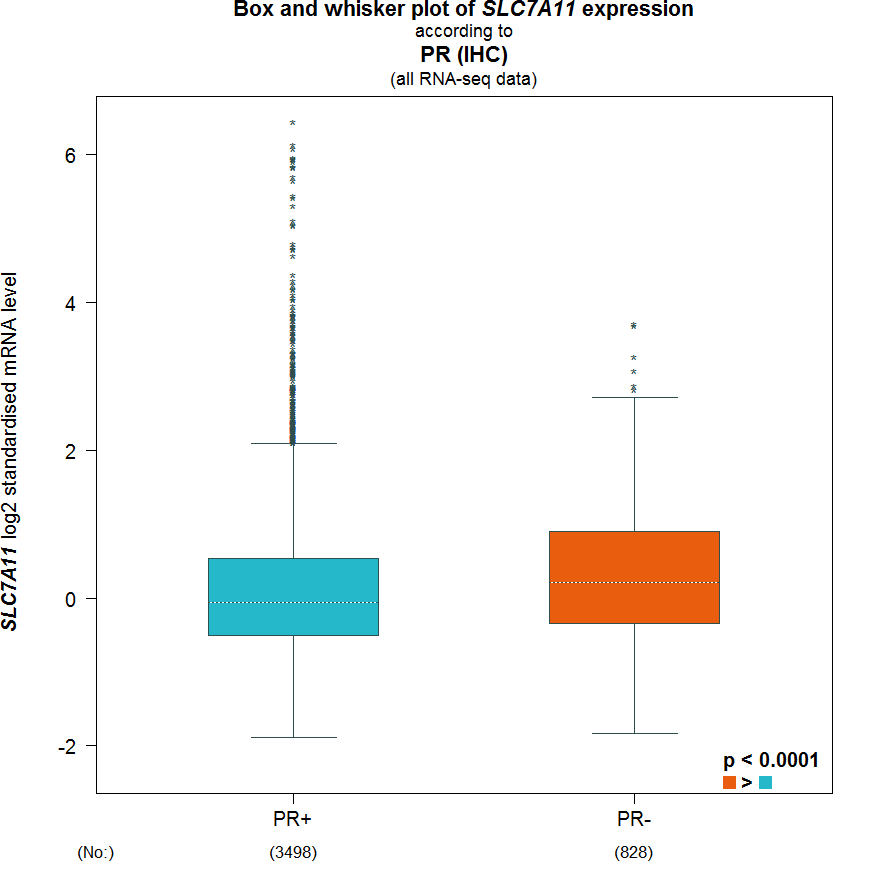

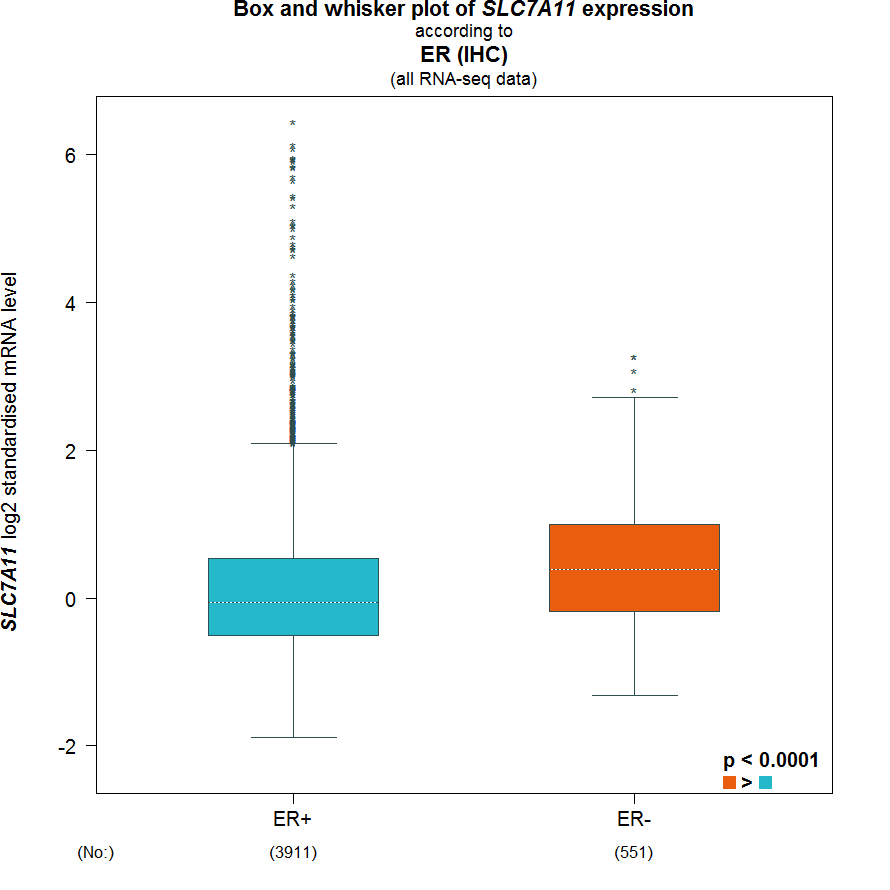

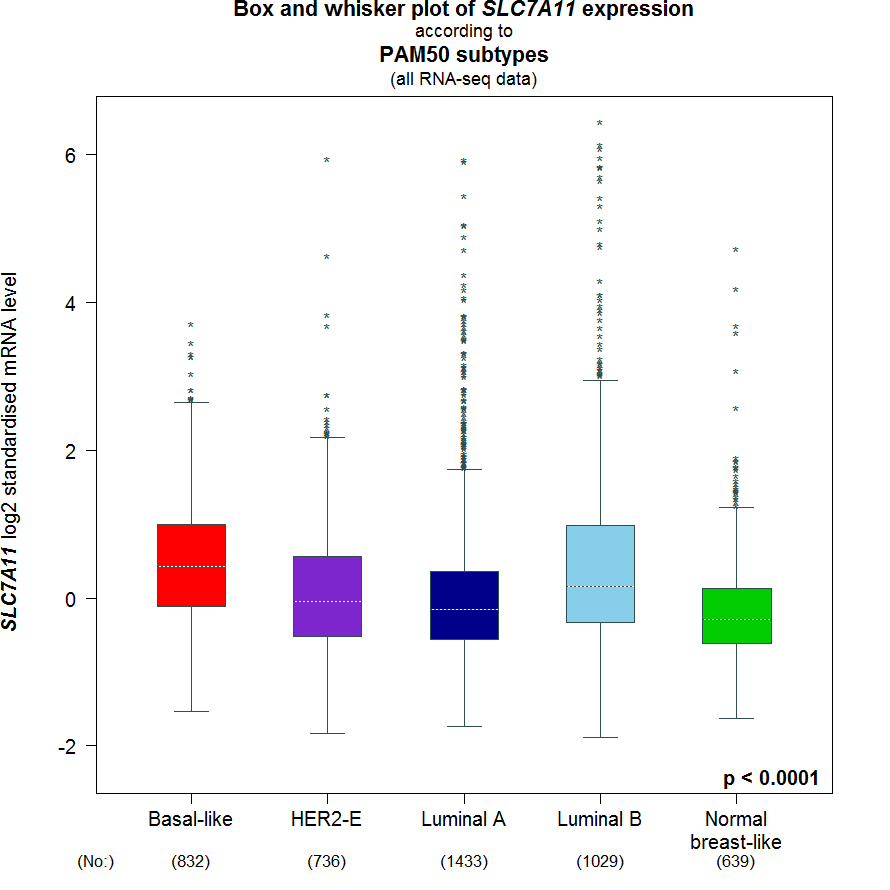

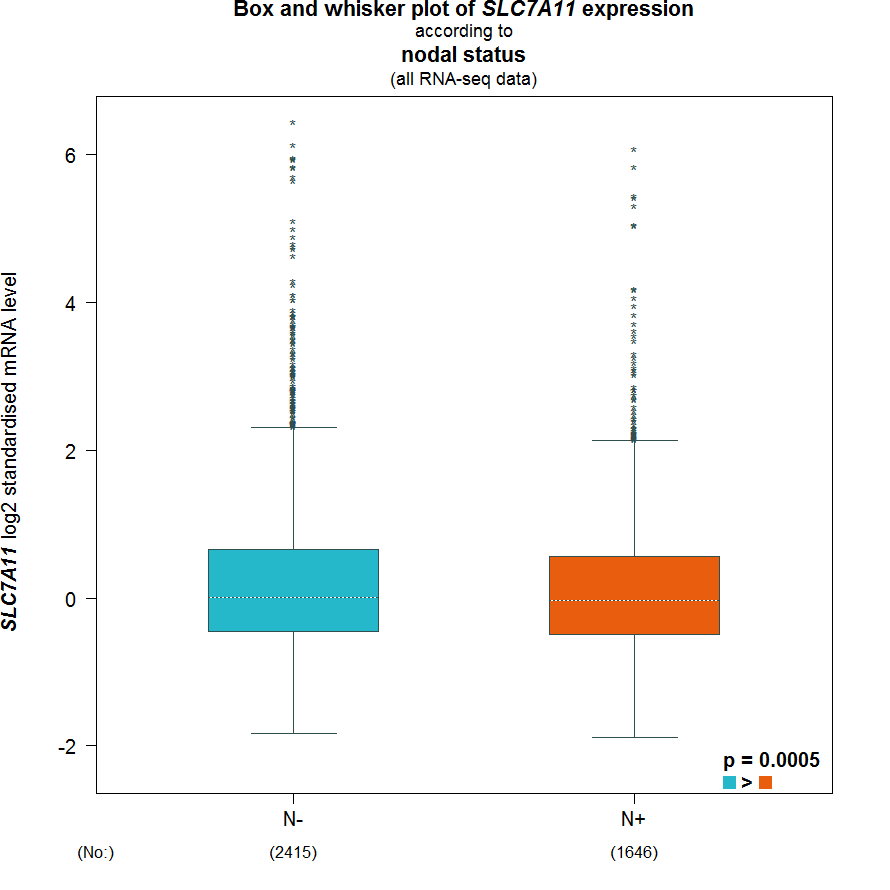

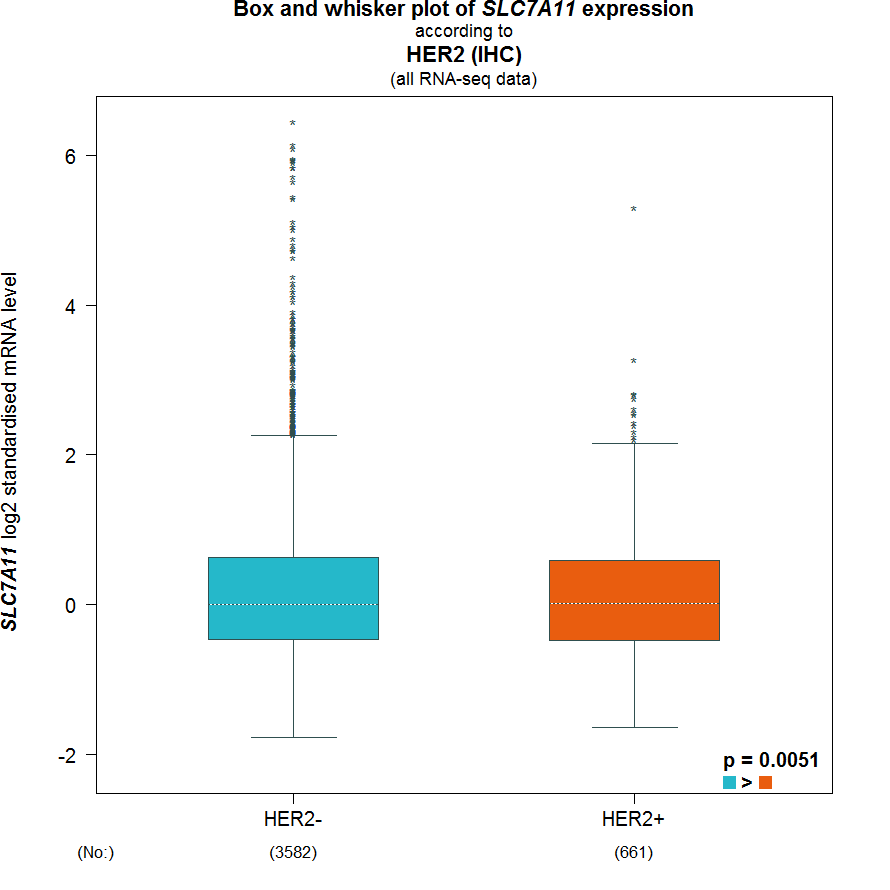


**Supplementary Figure 3S**

a

b

c

d

e

f

g

h


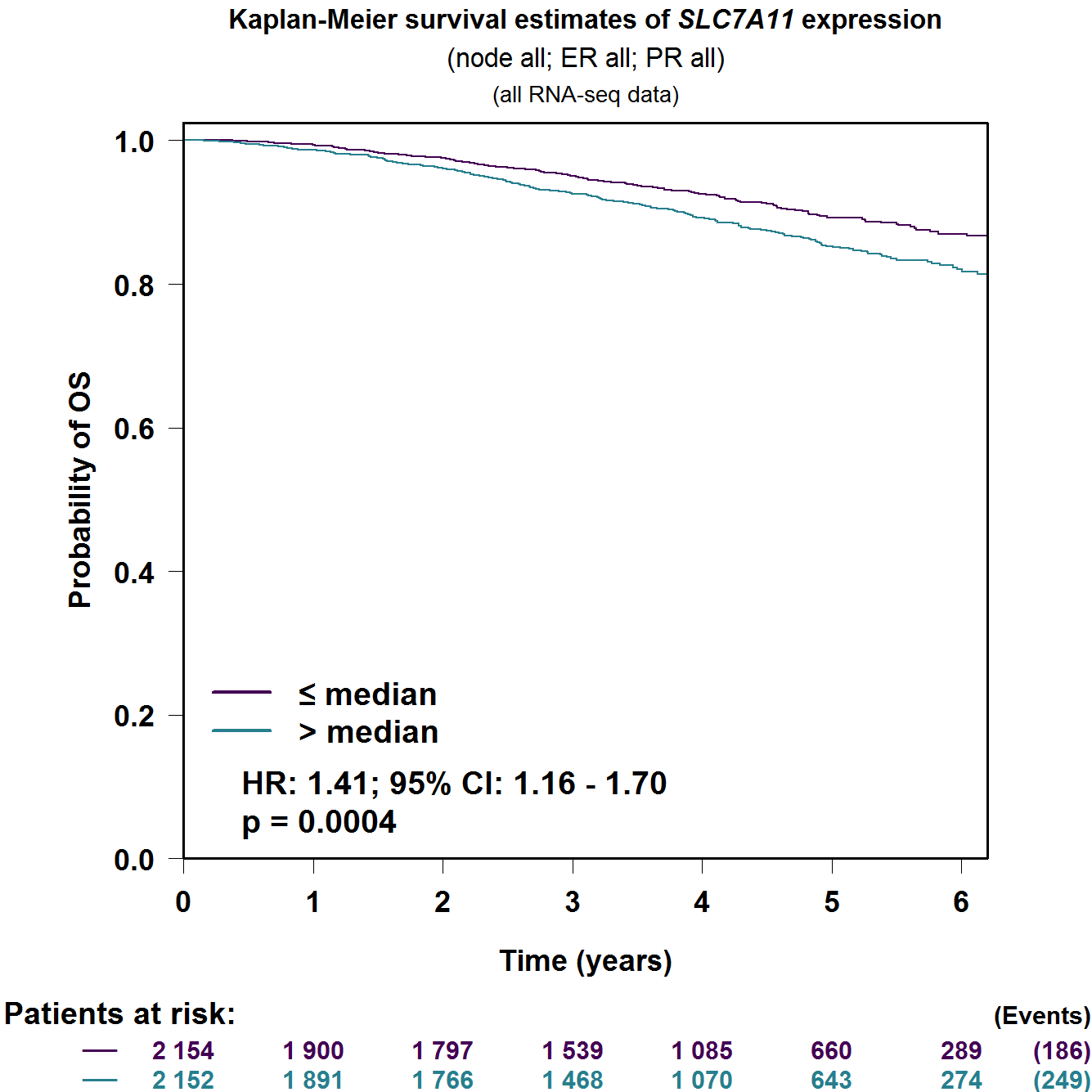

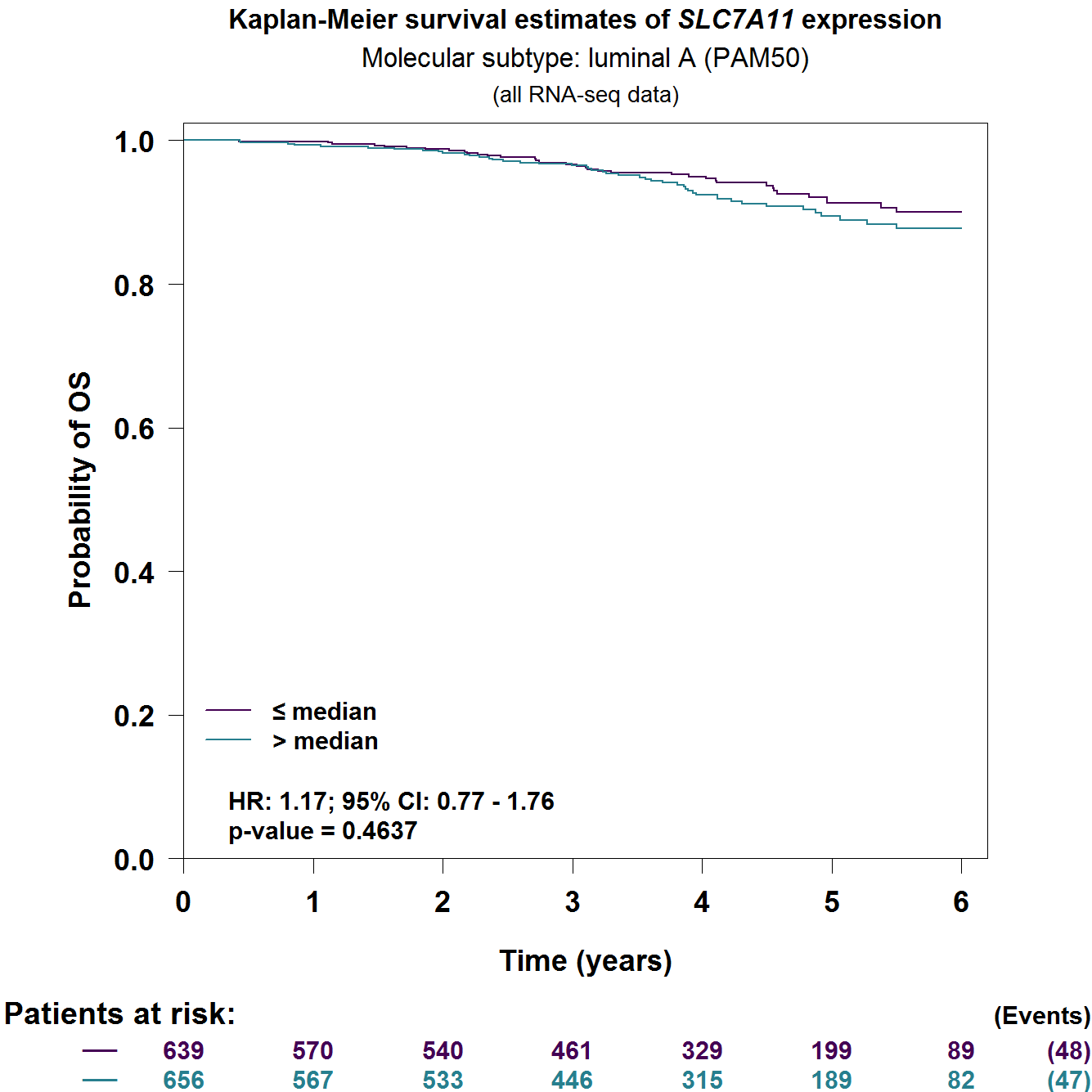


a

b


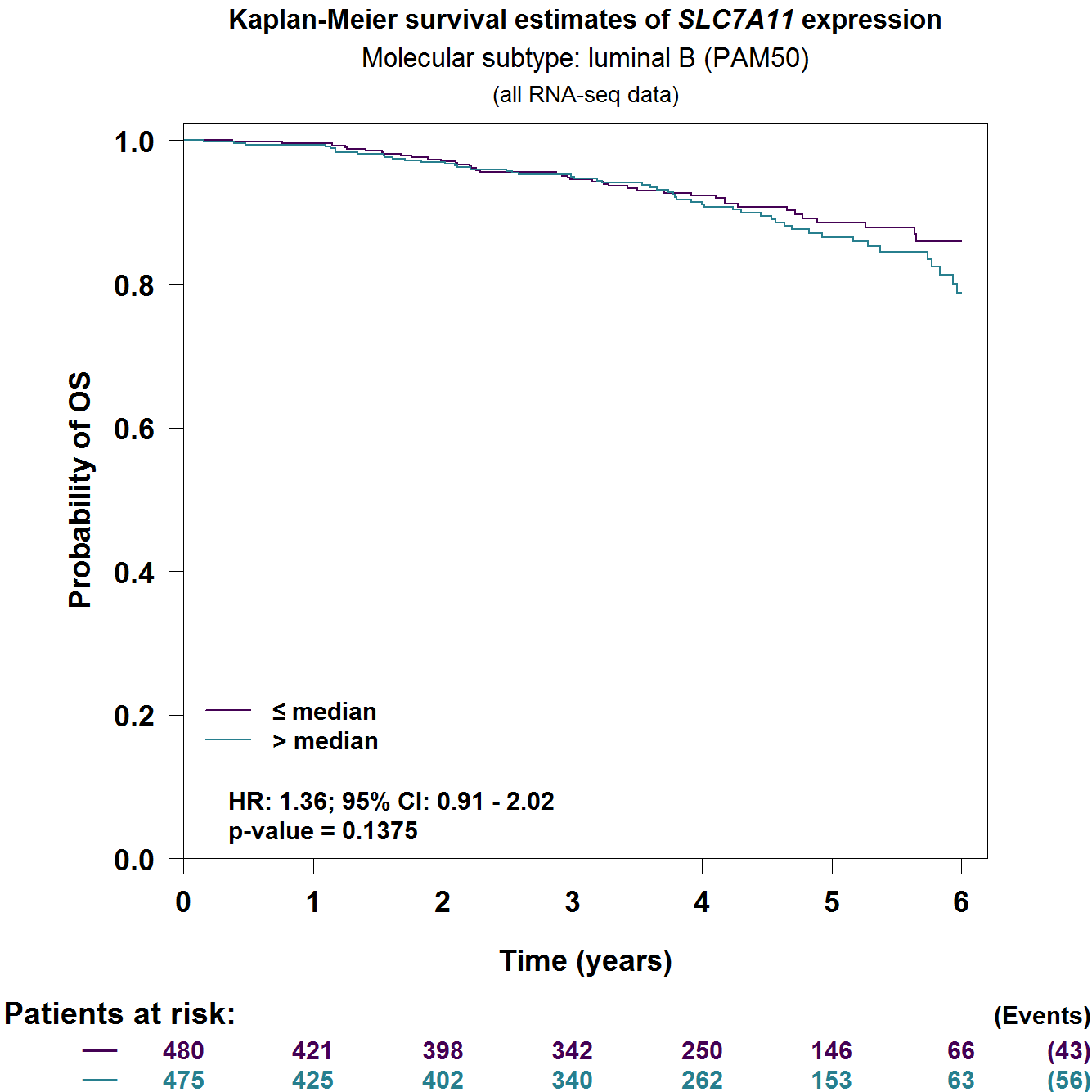

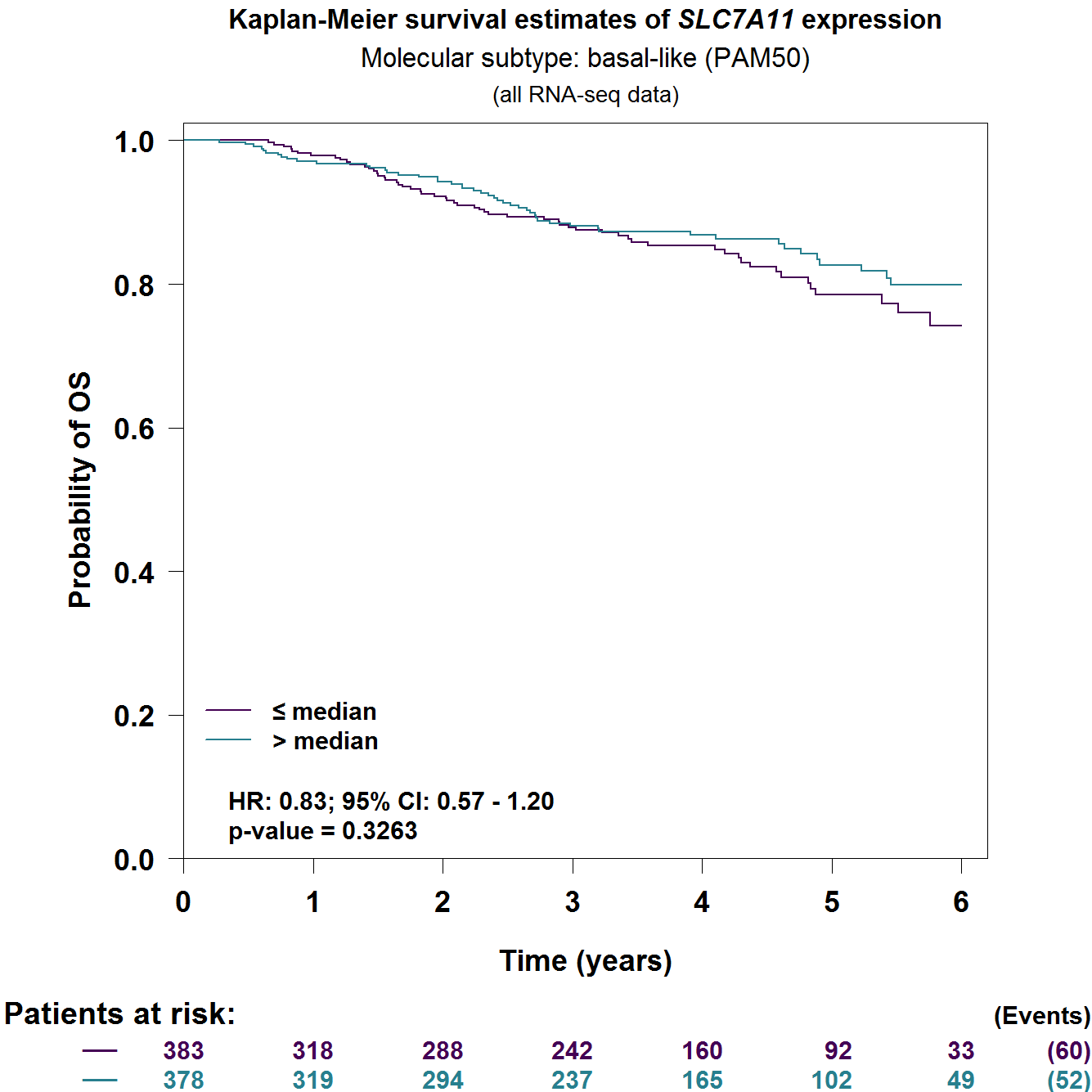


c

d


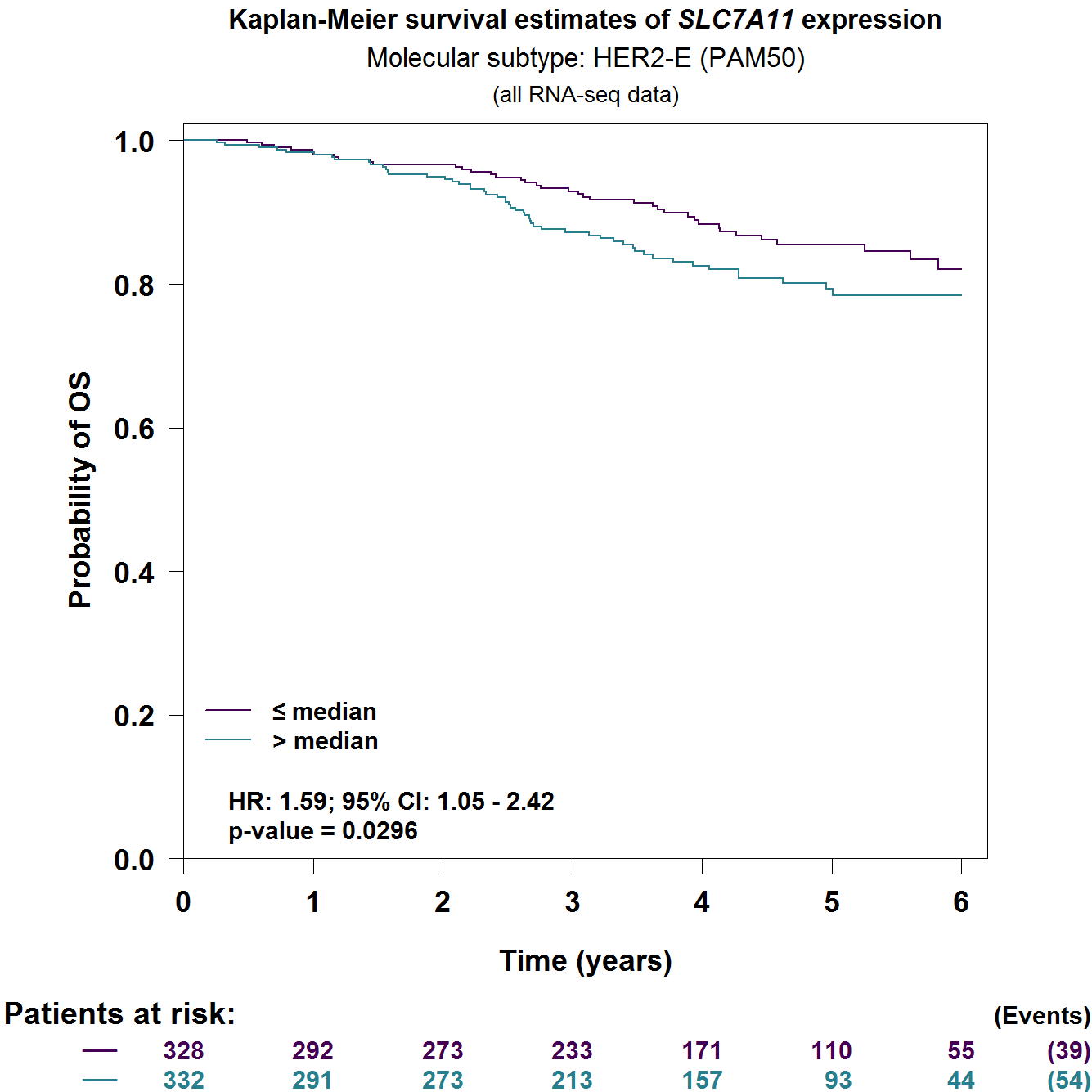

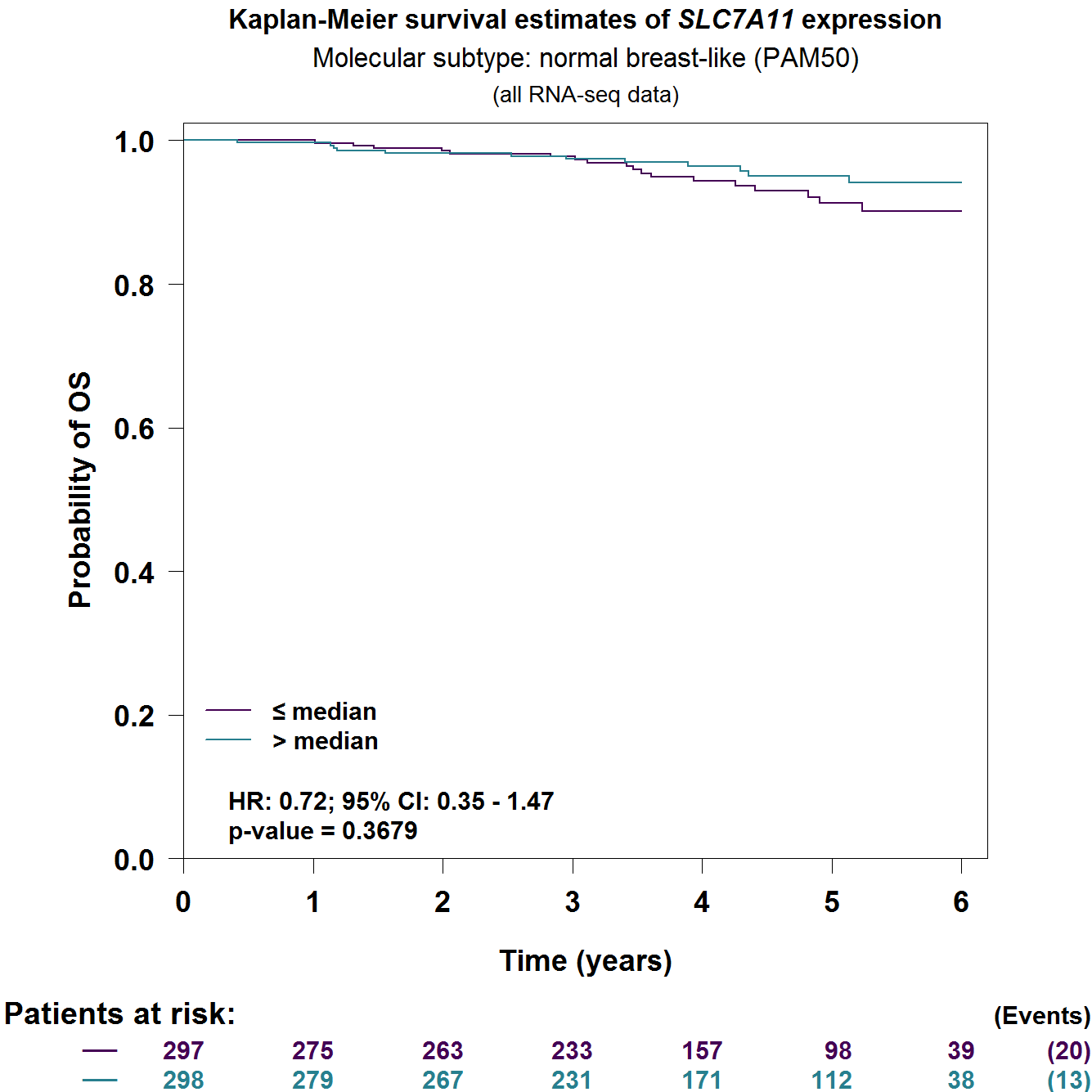


f

**Supplementary Figure 4S**

e


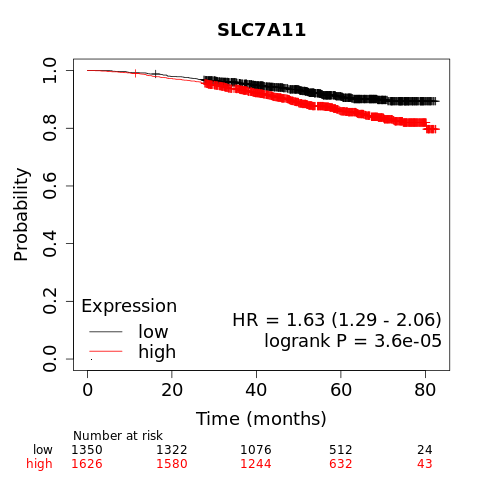


a

b


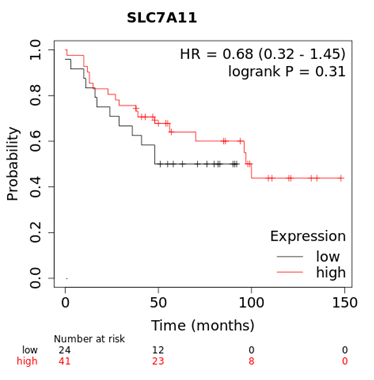


**Supplementary Figure 5S**
